# Trajectories of longitudinal biomarkers for mortality in severely burned patients

**DOI:** 10.1038/s41598-020-73286-8

**Published:** 2020-10-01

**Authors:** Jaechul Yoon, Dohern Kym, Jae Hee Won, Jun Hur, Haejun Yim, Yong Suk Cho, Wook Chun

**Affiliations:** 1 Department of Surgery and Critical Care, Burn Center, Hangang Sacred Heart Hospital, College of Medicine, Hallym University, 12, Beodeunaru-ro 7-gil, Youngdeungpo-gu, Seoul, 07247 Republic of Korea; 2 Graduate School of Medicine, Kangwon National University, Chuncheon, Republic of Korea

**Keywords:** Medical research, Risk factors

## Abstract

This study aimed to investigate the differences in the trajectory of blood biomarkers routinely assessed through forward- and backward-looking approaches among burn patients. This cohort study included patients above 18 years of age from February 2007 to December 2018. All the biomarkers were estimated from admission to discharge from the intensive care unit. Significant differences were observed in the platelet count at 40 days, prothrombin time (PT) at 32 days, white blood cell count at 26 days, creatinine levels at 22 days, and lactate and total bilirubin levels at 19 days before death. In reverse order, significant differences were observed in the fitted model in platelet count at 44 days and in the platelet count and PT at 33 days. We obtained more valuable information from the longitudinal biomarker trajectory using the backward-looking method than using the forward-looking method. The platelet count served as the earliest predictor of mortality among burn patients.

## Introduction

Burn injuries are one of most devastating forms of trauma and result in high morbidity and mortality. The mortality rate of burn patients has decreased since the last few decades due to advancements in critical care, nutritional support, antibiotics, and surgical procedures, such as excision and coverage with skin substitutes. However, the mortality rate of burn patients is still high compared to other critical care patients. Prediction of mortality and disease progression of critically ill patients in a systemic manner based on clear and objective data is an essential part of care in an Intensive Care Unit (ICU). Many biomarkers have been suggested and used to predict the outcome in critically ill and burn patients at certain time points, such as at the time of admission^1^, during acute kidney injury (AKI)^2^, or an intervention was conducted. The longitudinal biomarkers can give us more useful, appealing, and large amount of information because the repeated measurement of biomarkers over time allows physicians to get insights into the overall and individual biomarker trajectories^3^. The distribution of biomarkers over time can reflect the state of disease formation and help in earlier detection of the disease, thereby reducing the mortality associated with it^4^. Many routine biomarkers, including the white blood cell (WBC) count, lactate, creatinine, and total bilirubin (TB) levels, and prothrombin time (PT) are serially assessed to predict the disease progression and mortality in critically ill patients. Most longitudinal studies show biomarker trajectories sequentially from the initial treatment time. To observe the relationship between biomarkers and an event in a variety of ways, we looked over the biomarkers in reverse order of time until the development of the event by using event time as origin^5^. There are only a few studies that show the trajectory of the change of biomarkers in this manner. We hypothesized that such a reverse-time approach highlights differences that occur from temporal changes in the biomarkers, thus promptly reflecting disease progression patterns. Therefore, we aimed to investigate the clinical characteristics that are routinely measured in the blood and the forward and backward timepoints at which this difference occurs in burn patients.

## Results

### Baseline characteristics between survivors and non-survivors

A total of 2259 patients were included in this retrospective study and divided into the survival group with 1767 patients and non-survival group with 492 patients with the overall mortality being 21.8%. The overall median age was 48.0 years and higher in the non-survival group than the survival group (52.0 vs 46.0). Men were predominant in both the groups with an overall 81.9%. The overall median TBSA burned area was 24.0% and higher with 65.0% in the non-survival group. The prevalence of inhalation injury was significantly higher at 77.6% in the non-survival group. All severity scores were significantly higher (61.0 in APACHE IV, 160.0 in Hangang, 12.0 in ABSI) in the non-survival group. The duration of ICU stay was not significantly different between the two groups (*p* = 0.123) (Table 1). All baseline laboratory results herein were obtained upon admission and were found to significantly differ between the two groups (Table 1). The overall numbers of measurement for each biomarker were 40,671 for platelets and WBC, 37,157 for creatinine, 26,581 for TB, 24,526 for lactate, and 22,836 for PT. The number of subjects who were measured for biomarkers at the time of admission (day 0) in a forward manner were a total of 2243 for platelets and WBC, 2229 for creatinine, 2153 for lactate, 2227 for TB, and 2186 for PT. The number of subjects who were measured for biomarkers at the time of death or discharge (day 0) in a backward manner were a total of 2128 for platelets and WBC, 1931 for creatinine, 995 for lactate, 1388 for TB, and 929 for PT. The numbers of subjects measured for each biomarker at each day are summarized in Supplementary Table S1.

**Table 1:** Baseline characteristics between the two groups.

Variables	Survivors (n = 1767)	Non-survivors (n = 492)	Total (n = 2259)	*p* value
Age	46.0 [37.0;55.0]	52.0 [43.0;65.0]	48.0 [38.0;56.5]	< 0.001
**Gender**				0.455
Male	1454 (82.3%)	397 (80.7%)	1851 (81.9%)	
% TBSA	24.0 [14.0;37.0]	65.0 [40.0;85.0]	29.0 [17.0;48.0]	< 0.001
**Type**				< 0.001
FB	1213 (68.6%)	436 (88.6%)	1649 (73.0%)	
EB	317 (17.9%)	10 (2.0%)	327 (14.5%)	
SB	153 (8.7%)	33 (6.7%)	186 (8.2%)	
CoB	50 (2.8%)	10 (2.0%)	60 (2.7%)	
ChB	34 (1.9%)	3 (0.6%)	37 (1.6%)	
Inhalation	800 (45.3%)	382 (77.6%)	1182 (52.3%)	< 0.001
APACHE IV	33.0 [24.0;45.0]	61.0 [49.0;76.0]	38.0 [26.0;53.0]	< 0.001
Hangang	122.0 [113.0;132.0]	160.0 [149.0;176.0]	127.0 [115.0;144.0]	< 0.001
ABSI	7.0 [6.0; 9.0]	12.0 [10.0;14.0]	8.0 [6.0;10.0]	< 0.001
LOSICU	15.0 [6.0;34.0]	13.0 [7.5;24.0]	14.0 [6.0;32.0]	0.123
**Laboratory**				
Platelet	230.0 [184.0;280.0]	194.0 [134.0;272.0]	224.0 [173.0;278.5]	< 0.001
Lactate	2.6 [1.7; 4.0]	5.6 [3.9; 7.8]	3.0 [1.9; 5.0]	< 0.001
Creatinine	0.8 [0.6; 0.9]	1.0 [0.8; 1.4]	0.8 [0.7; 1.0]	< 0.001
TB	0.8 [0.5; 1.1]	1.1 [0.8; 1.7]	0.8 [0.6; 1.2]	< 0.001
PT	11.8 [10.9;12.9]	13.0 [11.7;14.8]	12.0 [11.0;13.3]	< 0.001
WBC	17.2 [12.9;22.6]	28.7 [20.5;36.8]	18.6 [13.7;26.0]	< 0.001

*n* number, *FB* Flame Burn, *SB* Scald Burn, *EB* Electrical Burn, *ChB* Chemical Burn, *CoB* Contact Burn, *% TBSA burned* percentage of total body surface area burned, *APACHE* Acute Physiology and Chronic Health Evaluation Score, *ABSI* Abbreviated Burn Severity Index, *LOSICU* length of intensive care unit stay, *TB* total bilirubin, *PT* prothrombin time, *WBC* white blood cell.

### Mixed effect logistic regression analysis for mortality

We analysed the known prognostic factors including age, TBSA, inhalation injury, and longitudinal biomarkers to confirm independent predictors of mortality, using a mixed effect logistic regression model. Age (odds ratio [OR]: 1.585), TBSA (OR: 1.766), and inhalation (OR: 19.326) were independent predictors of mortality regardless of the time factor and platelet count (OR: 0.9998) significantly varied over time (Table 2).

**Table 2:** Mixed effect logistic regression analysis for mortality.

Variables	Odds ratios	95% CI	*p* value
Age	1.585	1.451–1.731	< 0.001
% TBSA	1.766	1.632–1.912	< 0.001
Inhalation	19.326	2.951–126.572	0.002
Platelet: day	0.9998	0.9997–0.9999	0.004
Lactate: day	1.009	0.993–1.025	0.262
Creatinine: day	1.010	0.976–1.044	0.573
TB: day	1.002	0.993–1.012	0.659
PT: day	0.997	0.994–1.000	0.078
WBC: day	1.001	0.999–1.004	0.329

*CI* confidence interval, *% TBSA burned* percentage of total body surface area burned, *TB* total bilirubin, *PT* prothrombin time, *WBC* white blood cell. ‘[Variable]: day’ indicates the change in each variable over time.

### Trajectories for mean of longitudinal biomarkers between the survival and non-survival group

All biomarkers were plotted over time in a forward and backward manner with mean and confidence interval at each time. Platelet count in the forward manner was significantly higher at all time periods except on the day of admission; the mean platelet counts were 235.9 (232.0–239.8, 95% CI) in survivors and 221.8 (210.0–234.1, 95% CI) in non-survivors. The lactate level in the forward manner was significantly higher at all time points except on days 50, 51, and 53. Creatinine level was significantly higher within the first 26 days in the forward manner. The TB level was significantly higher within the first 38 days except day 5 in the forward manner. The PT in forward manner was significantly higher except on day 26, 38, 49, 54, and 55. The WBC count was significantly higher within the first 39 days except days 11, 12, and 14 in the forward manner.

**Figure 1:**
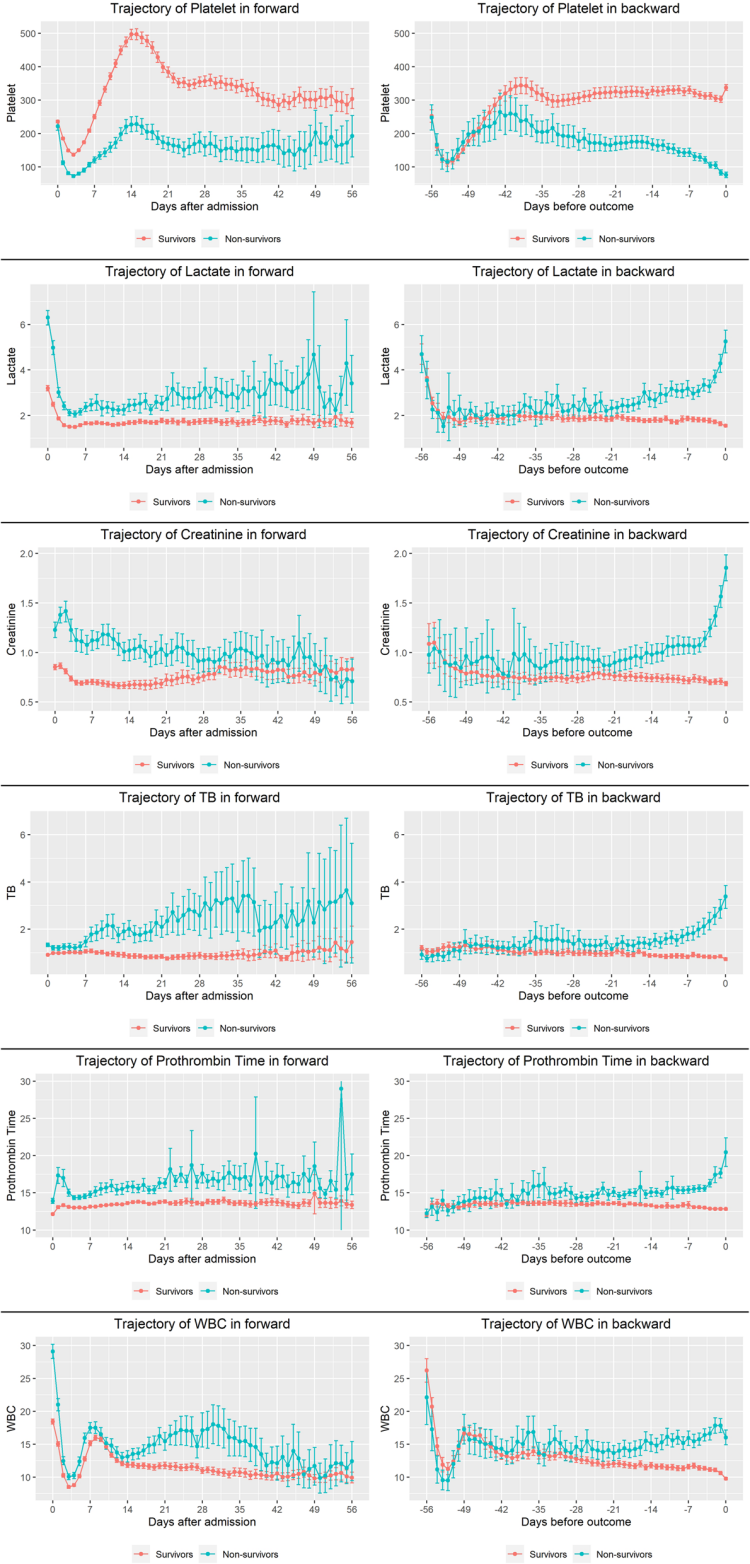
Trajectory of mean value for each biomarker between survivors and non-survivors. Error bar is 95% confidence interval calculated by bootstrap.

The significant difference in platelet count occurred from the 40 days before development of mortality and persisted till the end; the means were 340.9 (316.9–366.3, 95% CI) in survivors and 256.5 (206.5–306.5, 95% CI) in non-survivors. The statistically significant difference in backward manner persisted from 32 days for PT, 26 days for WBC, 22 days before death for creatinine, and 19 days for lactate and TB (Fig. 1). The mean and 95% CI for all biomarkers at each time points are summarized in Supplementary Table S1.

### Plot for the predictions over time of linear mixed effect model for each biomarker between survivors and non-survivors

**Figure 2:**
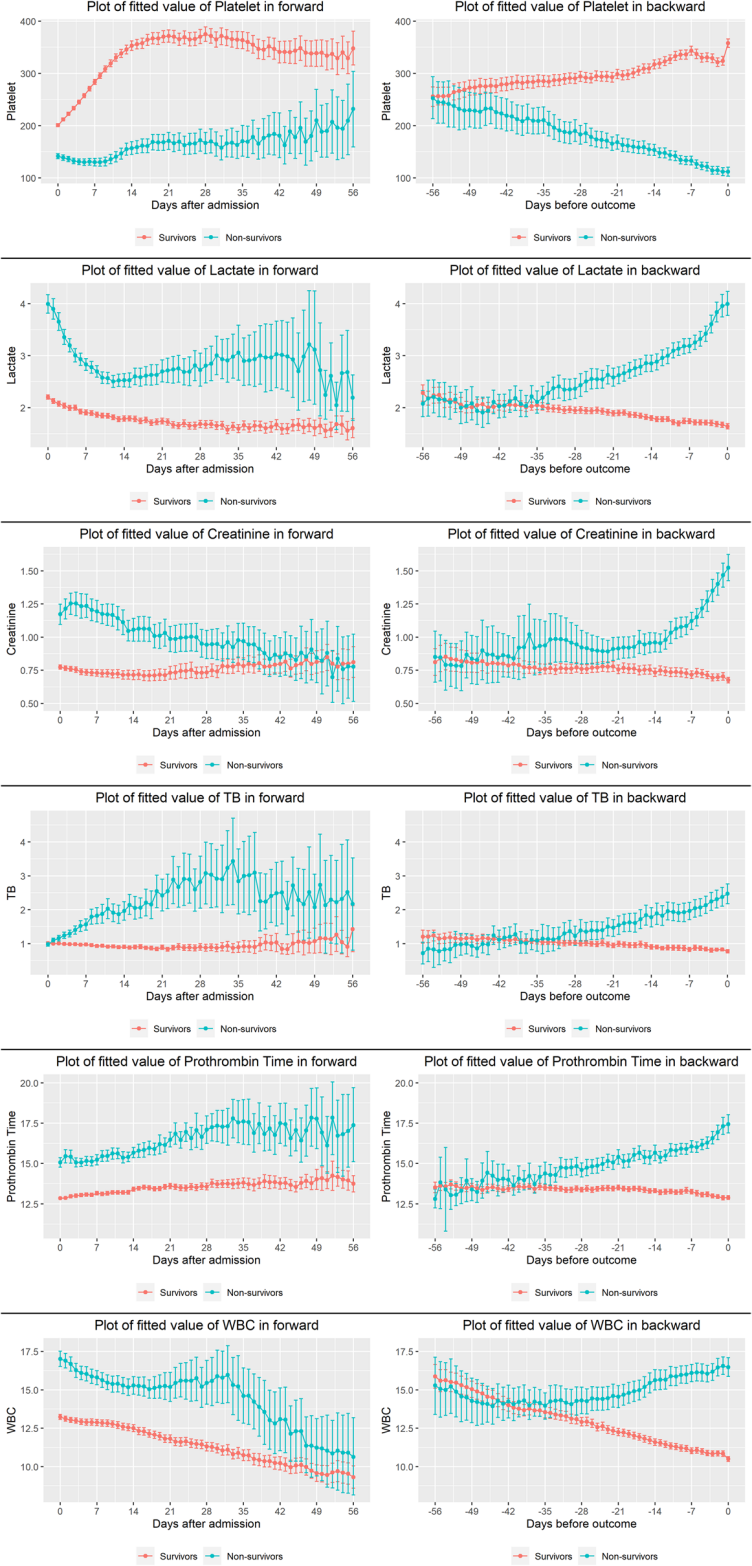
Plots over the time of predicted value using linear mixed effect model adjusted with risk factors, such as age, TBSA, and inhalation injury in forward and backward manner. Error bar is 95% confidence interval of predicted value.

All biomarkers were also plotted with predicted value using the linear mixed effect model, which was adjusted with known risk factors of burns (age, TBSA, inhalation) in both forward and backward manner. Fitted platelet count in forward manner were significantly higher at all time periods in survivors and the statistically significant difference was shown from 45 days before mortality in the backward manner. Fitted platelet count in forward manner were significantly higher at all time periods except on days 53 and 56 and significant changes were observed from 33 days before death in the reverse order. Fitted PT in the forward manner were significantly higher at all periods except on days 51, 53, and 54 and were significantly altered from 33 days before death in the reverse order. The significant difference of the fitted creatinine level was shown from 22 days before mortality. The significant difference of fitted TB level was shown from 25 days before mortality in reverse order. The fitted WBC count showed a significant difference from 44 days before death. The slopes of fitted WBC count in forward between the two groups showed a decreasing trend, but, increasing trend in non-survivors in backward manner (Fig. 2). The fitted value and 95% CI for all biomarkers at each time point are summarized in Supplementary Table S2.

## Discussion

Longitudinal biomarkers have been used to predict the outcome of interest because the change of biomarker within an individual can help improve the diagnostic accuracy. Most studies about the trajectory of longitudinal biomarkers look forward at them from a general origin time, such as the day of admission. In particular, for intensivists, looking at the biomarkers from the beginning plays an important role in predicting the diagnosis and prognosis, but it would be necessary to analyse them by rearranging all biomarkers, setting an event time as the origin, and then looking at the change of biomarkers prior to the event backwards. Huang et al.^3^ reported that backward-looking plots are much more informative than forward-looking plots to understand the shape of the trajectory leading up to the event of interest.

In the forward-looking manner, means of all biomarkers over time between two groups were almost clearly distinct for platelet, lactate, and PT, while being clearly distinct at the first half for creatinine, total bilirubin, and WBC (Fig. 1). We can infer that once patients were included in a certain group at the beginning, they remained in the group until the end. Therefore, it is difficult to obtain much information, because the conclusion was determined already. However, in the backward-looking manner, there was a statistically significant difference in the farthest 40 days before the occurrence of mortality for platelet and the shortest 19 days for TB and lactate. From these results, we can infer that platelet count could predict the mortality the earliest, although other confounding effects were not considered statistically. Clearly, both methods can bring in different information, and we thought that more clinically meaningful information could be derived from the backward-looking manner.

We also plotted the fitted value of each biomarker using mixed effect model adjusted with age, TBSA, and inhalation as known burn risk-factors and found the difference between the two manners (Fig. 2). The predicted values for all biomarkers except for creatinine were apparently different for almost all periods except the end period in the forward manner. For platelets, lactate, and PT, significant differences were observed between the two groups during all periods except for a few days, and a significant difference was observed in the platelet count at 44 days and in lactate and PT 33 days before death in the reverse order. These results indicate that the platelet count was the earliest predictor of mortality. Platelet count is a known risk factor and showed similar mean plots (Fig. 1) with other study showed platelet nadir at days 2–4, peak at days 12–17, and can differentiate survivors and non-survivors^6^. The significant difference in lactate levels was observed before 19 days in the simple mean plot but before 33 days before death in the fitted model because lactate reflects the severity of burns and the mortality risk^7^. For WBC count, a well-known predictor of sepsis and mortality among burn patients^8^, no difference was observed between the two groups in the forward manner (*p* = 0.537) and showed a decreasing trend, but we could infer that the resolution of leukocytosis is necessary to survive.

Creatinine, which is a known predictor of AKI, is a predictor of mortality in burns^9^. Total bilirubin, which is one of the risk factors in burns^10^, showed a similar pattern with creatinine in our study. The PT is also a known predictor of mortality in trauma patients, but is not a good predictor in burn patients^11^; however, the mean plot of PT (Fig. 1) in this study showed good differentiation between the two groups in both the manners. To predict an event, biomarker measurements over time are used presently. We could obtain a more significant amount of information from the longitudinal biomarker trajectory by looking backward than forward. Because platelets showed discrete differences between the two groups, the forward manner may give the perception that the patients have already taken a path (to die or stay alive) before even receiving treatment. However, we can identify the time at which the two groups diverge, so that clinicians can get a better perception of the time to death or the risk of death, thereby becoming more actively involved in the treatment.

The limitations of our study are as follows. First, laboratory values were measured less regularly in the survivor group toward the discharge from the ICU. Further, the values showed broader and irregular confidence intervals towards the end of the analysis period in the forward-looking manner and towards the start in the backward-looking manner, which can be explained by the reduced number of patients/available data at these respective timepoints. To overcome these limitations resulting from unbalanced data, we used a linear mixed effect model. Second, this study did not primarily aim to determine the risk factors through routine statistical analysis including the mixed effect logistic analysis model and extended Cox survival analysis, indicating a method of data analysis in the reverse order; considering the retrospective nature of the study, the clinical applications of our results are limited. The main strength of this study is the introduction of a new backward-looking manner to find the changing pattern of predictors over time, which cannot be found by the commonly used forward-looking manner.

Through the simple mean plot of platelet count by the backward method, platelet count could aid in prediction of mortality 40 days earlier in burn victims. Considering the burn risk factors, such as age, TBSA, and smoke inhalation, platelet count independently predicted mortality 45 days before the event.

## Methods

### Patients

This retrospective cohort study included patients aged more than 18 years who were admitted within 24 h after a burn injury in the burn intensive care unit (BICU) of Hangang Sacred Heart Hospital, Hallym University Medical Center from February 2007 to December 2018. The criteria for admission to the BICU were as follows: (1) partial thickness burn of more than 20% of total body surface area (TBSA) among adults and more than 10% of TBSA among patients aged above 65 years, (2) inhalation injury, (3) electrical burn, (4) pre-existing medical disorder potentially resulting in complications or affecting mortality, and (5) concomitant trauma potentially elevating the morbidity and mortality risks. Clinical longitudinal parameters known as predictors, including the WBC count, platelet count, lactate^7^, creatinine, TB, and PT were retrieved from a clinical database warehouse at Hangang Sacred Heart Hospital. All variables were collected from the time of admission to death in the non-survival group and to discharge from the ICU in the survival group. When the biomarkers were measured several times a day, we collected the worst value of biomarkers. We recorded the demographic characteristics of patients, including age, sex, and TBSA, which were determined using a modified Lund and Browder chart^12^. Furthermore, we obtained information regarding the type of burn, duration of ICU stay, and the presence of inhalation injury defined by the history of smoke exposure in a closed space and prolonged extrication, and/or physical findings, such as singed facial hair, carbonaceous deposits in the oropharynx or sputum, facial burns, and voice changes^13^. The primary outcome was in-hospital 56-day mortality. The severity of injury was reported using the Abbreviated Burn Severity Index (ABSI)^14^, and the newly developed Hangang score^15^ in our centre, and Acute Physiology and Chronic Health Evaluation Score (APACHE) IV^16^. This study was performed in accordance with the tenets of the Declaration of Helsinki and approved by the Institutional Review Board (IRB) of Hangang Sacred Heart Hospital. Informed consent was waived by IRB of Hangang Sacred Heart Hospital, considering the retrospective nature of the study without any intervention.

### Treatment strategy

Wound dressing was performed every day with appropriate pain management. In the absence of signs of infection, no dressing material containing antimicrobial agents was used and a simple wet dressing was performed in such cases. Initial burn wound excision with allograft application was carried out 5 days after burn injury, followed by autografting, if required on the basis of the patient’s condition. To treat systemic infection, antibiotics were prescribed on the basis of the results of sputum, blood, urine, and wound cultures.

### Statistical analysis

Baseline demographic characteristics were reported as follows. Continuous variables distributed normally were presented as means ± standard deviation (SD) or distributed non-normally as medians (25th interquartile rage [IQR]–75th IQR). The paired *t* test or Wilcoxon signed rank test depending on normality was used to determine the differences between two groups. Categorical variables are presented as percentages and were analysed by the Chi-square test. To confirm independent predictors of mortality using unbalanced longitudinal biomarkers which were not measured at a common period, we used mixed effect logistic regression models using lme4 packages of R-project program and to analyse the patterns of unbalanced longitudinal biomarkers, we used linear mixed effect models using nlme packages and determined the confidence interval of differences at each time point between two groups using bootstraps. Linear mixed models of each biomarker were adjusted with age, TBSA, and the presence of inhalation injury as known risk factors for burns. Plots were described in two ways. One is in the forward manner in which the biomarker was looked over sequentially from admission as origin. The other is the backward manner in which the biomarker was looked over in reverse order of time until the development of event (mortality in this study) by using the day of mortality as the origin for non-survivors and the day of discharge of the ICU as the origin for survivors. Two side *p* value < 0.05 were considered statistically significant. All analyses were conducted using the statistical R-project program version 3.6.2. (R Core Team (2019). R: A language and environment for statistical computing. R Foundation for Statistical Computing, Vienna, Austria. URL https://www.R-project.org/).

## Supplementary information

Supplementary Table S1.

Supplementary Table S2.
