# Supplementary material for: Trajectories of longitudinal biomarkers for mortality in severely burned patients
Source: Sci Rep. 2020 Oct 1;10:16193. doi: 10.1038/s41598-020-73286-8 (PMC7530734; doi:10.1038/s41598-020-73286-8)
Supplement: Supplementary file 1 — Supplementary Table S1. [file 41598_2020_73286_MOESM1_ESM.docx]

**Trajectories of longitudinal biomarkers for mortality in severely burned patients**

Running title: Trajectories of longitudinal biomarkers in burns

Jaechul Yoon^1,2^, Dohern Kym^1+^, Jae Hee Won^1^, Jun Hur^1^*, Haejun Yim^1^, Yong Suk Cho^1^, Wook Chun^1^

^1^Department of Surgery and Critical Care, Burn Center, Hangang Sacred Heart Hospital, College of Medicine, Hallym University Medical, Seoul, Republic of Korea

^2^ Graduate school of Medicine, Kanwon National University, Chuncheon, Republic of Korea

^+^ This author contributed equally to this work as a first author. ORCID: 0000-0001-9178-0599

*Corresponding authors

Department of Surgery and Critical Care, Burn Center, Hangang Sacred Heart Hospital, College of Medicine, Hallym University **12, Beodeunaru-ro 7-gil**, Youngdeungpo-gu, Seoul, Korea, 07247

Tel. 82-2-2639-5446, Fax. 82-2-2678-4386, E-mail: [hammerj@hallym.or.kr](mailto:hammerj@hallym.or.kr)

Supplementary Table S1

1.The number of measurements, mean, 95% confidence interval in forward manner

A) Platelet, Lactate, Creatinine in forward manner

| Days | Group | Platelet | | | lactate | | | creatinine | | |
| --- | --- | --- | --- | --- | --- | --- | --- | --- | --- | --- |
|  |  | N | mean | 95% CI | N | mean | 95% CI | N | mean | 95% CI |
| 0 | Survivors | 1753 | 235.9 | 232~239.8 | 1675 | 3.19 | 3.08~3.31 | 1743 | 0.85 | 0.83~0.88 |
| 0 | Non-survivors | 490 | 221.8 | 210~234.1 | 478 | 6.30 | 5.98~6.62 | 486 | 1.23 | 1.15~1.3 |
| 1 | Survivors | 1730 | 185.3 | 182.2~188.6 | 1304 | 2.49 | 2.4~2.57 | 1677 | 0.86 | 0.84~0.89 |
| 1 | Non-survivors | 491 | 112.6 | 107~118.3 | 418 | 4.97 | 4.67~5.29 | 487 | 1.38 | 1.3~1.46 |
| 2 | Survivors | 1619 | 149.1 | 146~152 | 970 | 1.86 | 1.79~1.93 | 1496 | 0.81 | 0.79~0.84 |
| 2 | Non-survivors | 482 | 81.3 | 77~85.6 | 366 | 3.01 | 2.8~3.22 | 461 | 1.42 | 1.31~1.52 |
| 3 | Survivors | 1448 | 137.0 | 133.9~140.4 | 859 | 1.56 | 1.51~1.62 | 1329 | 0.74 | 0.71~0.76 |
| 3 | Non-survivors | 452 | 72.8 | 68.6~77 | 323 | 2.41 | 2.24~2.59 | 434 | 1.23 | 1.11~1.34 |
| 4 | Survivors | 1360 | 150.2 | 146.6~153.8 | 774 | 1.50 | 1.45~1.56 | 1231 | 0.70 | 0.67~0.72 |
| 4 | Non-survivors | 428 | 80.4 | 75.8~85 | 307 | 2.12 | 1.99~2.25 | 412 | 1.12 | 1.01~1.24 |
| 5 | Survivors | 1242 | 173.7 | 169.4~177.7 | 707 | 1.49 | 1.44~1.55 | 1123 | 0.69 | 0.67~0.71 |
| 5 | Non-survivors | 404 | 90.6 | 84.8~96.2 | 268 | 2.06 | 1.93~2.19 | 376 | 1.11 | 1~1.23 |
| 6 | Survivors | 1147 | 208.8 | 203.8~213.7 | 641 | 1.57 | 1.51~1.63 | 1036 | 0.70 | 0.68~0.72 |
| 6 | Non-survivors | 380 | 106.9 | 99.9~113.5 | 249 | 2.16 | 2.02~2.3 | 354 | 1.08 | 0.98~1.17 |
| 7 | Survivors | 1071 | 250.2 | 244~256.4 | 591 | 1.65 | 1.59~1.72 | 961 | 0.70 | 0.68~0.73 |
| 7 | Non-survivors | 362 | 121.1 | 112.3~130 | 235 | 2.37 | 2.17~2.56 | 327 | 1.12 | 1.02~1.22 |
| 8 | Survivors | 1003 | 292.0 | 284.2~299.3 | 542 | 1.64 | 1.58~1.71 | 901 | 0.70 | 0.67~0.72 |
| 8 | Non-survivors | 340 | 133.7 | 123.2~144.9 | 217 | 2.46 | 2.2~2.72 | 307 | 1.12 | 1.04~1.21 |
| 9 | Survivors | 952 | 332.7 | 323.9~341.1 | 536 | 1.68 | 1.6~1.75 | 841 | 0.69 | 0.66~0.72 |
| 9 | Non-survivors | 316 | 143.6 | 130.8~156.5 | 198 | 2.57 | 2.22~2.92 | 288 | 1.18 | 1.08~1.28 |
| 10 | Survivors | 903 | 371.2 | 361.3~381.4 | 502 | 1.66 | 1.59~1.73 | 800 | 0.68 | 0.65~0.71 |
| 10 | Non-survivors | 290 | 159.3 | 144.5~173.8 | 180 | 2.31 | 1.98~2.64 | 268 | 1.18 | 1.07~1.29 |
| 11 | Survivors | 873 | 409.2 | 397.4~420.4 | 486 | 1.63 | 1.56~1.69 | 777 | 0.67 | 0.64~0.7 |
| 11 | Non-survivors | 265 | 172.7 | 155.9~188.5 | 163 | 2.36 | 2.11~2.59 | 244 | 1.14 | 1.03~1.24 |
| 12 | Survivors | 831 | 448.8 | 435.9~462 | 462 | 1.58 | 1.51~1.65 | 733 | 0.66 | 0.63~0.7 |
| 12 | Non-survivors | 233 | 198.5 | 179.3~217 | 144 | 2.27 | 1.95~2.58 | 211 | 1.08 | 0.97~1.2 |
| 13 | Survivors | 796 | 474.6 | 459.8~488.4 | 433 | 1.61 | 1.53~1.68 | 704 | 0.67 | 0.64~0.7 |
| 13 | Non-survivors | 219 | 221.0 | 200~241.9 | 138 | 2.24 | 2.07~2.41 | 202 | 1.01 | 0.92~1.1 |
| 14 | Survivors | 761 | 496.7 | 480.8~511.5 | 416 | 1.63 | 1.55~1.7 | 678 | 0.67 | 0.64~0.71 |
| 14 | Non-survivors | 201 | 227.3 | 205.3~250.1 | 127 | 2.25 | 2.02~2.49 | 183 | 1.02 | 0.92~1.13 |
| 15 | Survivors | 740 | 496.5 | 480.7~513.1 | 403 | 1.68 | 1.6~1.76 | 659 | 0.68 | 0.64~0.72 |
| 15 | Non-survivors | 193 | 228.3 | 206.5~251.3 | 124 | 2.44 | 2.17~2.71 | 172 | 1.04 | 0.93~1.14 |
| 16 | Survivors | 699 | 487.2 | 469.2~503.6 | 385 | 1.69 | 1.6~1.77 | 632 | 0.67 | 0.63~0.72 |
| 16 | Non-survivors | 179 | 221.2 | 197.8~244.9 | 111 | 2.47 | 2.23~2.71 | 159 | 1.06 | 0.94~1.18 |
| 17 | Survivors | 700 | 477.4 | 460.4~493.5 | 370 | 1.74 | 1.64~1.84 | 621 | 0.67 | 0.62~0.72 |
| 17 | Non-survivors | 168 | 205.2 | 182.9~227.2 | 105 | 2.52 | 2.11~2.94 | 154 | 1.02 | 0.9~1.12 |
| 18 | Survivors | 670 | 457.3 | 440.4~474.4 | 375 | 1.71 | 1.64~1.79 | 595 | 0.67 | 0.63~0.72 |
| 18 | Non-survivors | 160 | 204.8 | 181.8~227.5 | 97 | 2.63 | 2.28~2.97 | 142 | 0.96 | 0.86~1.06 |
| 19 | Survivors | 637 | 428.0 | 412.4~444.1 | 336 | 1.69 | 1.61~1.77 | 564 | 0.69 | 0.64~0.74 |
| 19 | Non-survivors | 148 | 187.0 | 167.1~207.4 | 87 | 2.26 | 2.04~2.48 | 135 | 1.00 | 0.88~1.11 |
| 20 | Survivors | 630 | 398.1 | 382.9~412.4 | 339 | 1.69 | 1.6~1.78 | 580 | 0.68 | 0.64~0.73 |
| 20 | Non-survivors | 144 | 174.3 | 154.6~192.9 | 100 | 2.59 | 2.32~2.88 | 133 | 1.03 | 0.89~1.18 |
| 21 | Survivors | 592 | 384.0 | 368.4~399.3 | 326 | 1.78 | 1.68~1.88 | 532 | 0.72 | 0.67~0.78 |
| 21 | Non-survivors | 137 | 169.5 | 150.5~189.2 | 91 | 2.52 | 2.22~2.84 | 122 | 0.98 | 0.86~1.09 |
| 22 | Survivors | 577 | 366.3 | 351.7~380.2 | 301 | 1.72 | 1.63~1.82 | 519 | 0.72 | 0.66~0.77 |
| 22 | Non-survivors | 128 | 164.5 | 144.2~185 | 84 | 2.73 | 2.2~3.27 | 116 | 1.00 | 0.87~1.13 |
| 23 | Survivors | 538 | 351.2 | 337.6~365 | 281 | 1.75 | 1.63~1.88 | 481 | 0.74 | 0.68~0.8 |
| 23 | Non-survivors | 124 | 162.0 | 140.2~183 | 84 | 3.16 | 2.41~3.87 | 112 | 1.05 | 0.92~1.2 |
| 24 | Survivors | 515 | 353.0 | 338.3~367.2 | 274 | 1.68 | 1.58~1.79 | 461 | 0.76 | 0.69~0.82 |
| 24 | Non-survivors | 113 | 151.9 | 130.1~174.4 | 74 | 2.94 | 2.46~3.42 | 105 | 1.05 | 0.9~1.19 |
| 25 | Survivors | 483 | 344.8 | 330~359.3 | 265 | 1.76 | 1.66~1.88 | 444 | 0.76 | 0.7~0.82 |
| 25 | Non-survivors | 101 | 161.0 | 136.9~185.4 | 61 | 2.76 | 2.28~3.25 | 93 | 0.99 | 0.86~1.12 |
| 26 | Survivors | 470 | 348.5 | 334.1~362.3 | 245 | 1.66 | 1.56~1.76 | 416 | 0.73 | 0.68~0.77 |
| 26 | Non-survivors | 97 | 168.9 | 140.9~197.7 | 58 | 2.77 | 2.34~3.22 | 91 | 0.98 | 0.83~1.12 |
| 27 | Survivors | 464 | 354.8 | 340~369.3 | 237 | 1.71 | 1.6~1.82 | 413 | 0.74 | 0.7~0.79 |
| 27 | Non-survivors | 91 | 175.7 | 145.3~204.9 | 56 | 2.76 | 2.3~3.22 | 82 | 0.92 | 0.79~1.04 |
| 28 | Survivors | 445 | 356.9 | 341.3~372.4 | 219 | 1.74 | 1.62~1.86 | 408 | 0.76 | 0.71~0.81 |
| 28 | Non-survivors | 84 | 161.8 | 132.8~190.2 | 53 | 2.88 | 2.28~3.46 | 80 | 0.92 | 0.8~1.04 |
| 29 | Survivors | 415 | 360.3 | 343.3~377.3 | 218 | 1.75 | 1.64~1.86 | 375 | 0.78 | 0.72~0.84 |
| 29 | Non-survivors | 80 | 169.6 | 137.3~205.2 | 50 | 3.19 | 2.3~4.08 | 75 | 0.93 | 0.81~1.05 |
| 30 | Survivors | 404 | 350.7 | 333.7~367.6 | 215 | 1.75 | 1.64~1.87 | 373 | 0.78 | 0.72~0.84 |
| 30 | Non-survivors | 75 | 161.4 | 130.5~193.5 | 50 | 2.79 | 2.34~3.24 | 73 | 0.91 | 0.78~1.02 |
| 31 | Survivors | 379 | 354.7 | 335.8~372.3 | 196 | 1.78 | 1.68~1.88 | 342 | 0.85 | 0.78~0.93 |
| 31 | Non-survivors | 73 | 148.9 | 116~179.7 | 48 | 3.13 | 2.56~3.67 | 69 | 0.93 | 0.8~1.05 |
| 32 | Survivors | 364 | 347.0 | 329.3~365 | 183 | 1.69 | 1.56~1.81 | 334 | 0.85 | 0.77~0.92 |
| 32 | Non-survivors | 70 | 154.8 | 122.5~188.1 | 46 | 3.00 | 2.42~3.62 | 67 | 0.99 | 0.84~1.13 |
| 33 | Survivors | 353 | 347.1 | 329.6~364.7 | 173 | 1.70 | 1.55~1.85 | 322 | 0.82 | 0.75~0.9 |
| 33 | Non-survivors | 67 | 156.4 | 121.8~191.4 | 43 | 2.84 | 2.2~3.49 | 65 | 0.96 | 0.81~1.11 |
| 34 | Survivors | 329 | 340.5 | 321.8~359 | 172 | 1.75 | 1.62~1.88 | 303 | 0.83 | 0.76~0.9 |
| 34 | Non-survivors | 59 | 147.1 | 118.1~177.6 | 42 | 2.94 | 2.31~3.59 | 55 | 1.03 | 0.85~1.2 |
| 35 | Survivors | 330 | 343.5 | 325.2~362 | 170 | 1.64 | 1.52~1.77 | 293 | 0.82 | 0.75~0.9 |
| 35 | Non-survivors | 56 | 153.4 | 118~189 | 35 | 2.76 | 2.16~3.33 | 51 | 1.04 | 0.87~1.21 |
| 36 | Survivors | 297 | 330.4 | 311.2~350 | 140 | 1.71 | 1.57~1.85 | 261 | 0.85 | 0.77~0.93 |
| 36 | Non-survivors | 54 | 152.8 | 113.8~189.4 | 39 | 3.16 | 2.31~3.95 | 53 | 1.02 | 0.84~1.2 |
| 37 | Survivors | 285 | 332.5 | 312.8~353 | 154 | 1.70 | 1.58~1.81 | 260 | 0.83 | 0.76~0.9 |
| 37 | Non-survivors | 54 | 152.6 | 119~186.6 | 39 | 3.07 | 2.42~3.72 | 50 | 0.99 | 0.8~1.17 |
| 38 | Survivors | 254 | 316.0 | 295.8~336.5 | 132 | 1.77 | 1.6~1.92 | 227 | 0.84 | 0.76~0.92 |
| 38 | Non-survivors | 48 | 149.2 | 113.8~184.9 | 35 | 3.20 | 1.98~4.37 | 46 | 0.94 | 0.72~1.16 |
| 39 | Survivors | 264 | 304.1 | 283.9~323 | 140 | 1.83 | 1.69~1.98 | 243 | 0.82 | 0.75~0.89 |
| 39 | Non-survivors | 43 | 160.6 | 115.5~202.7 | 32 | 2.82 | 2.12~3.51 | 37 | 0.97 | 0.69~1.23 |
| 40 | Survivors | 246 | 301.6 | 282.1~319.8 | 126 | 1.71 | 1.55~1.87 | 224 | 0.81 | 0.73~0.88 |
| 40 | Non-survivors | 46 | 163.0 | 122.6~206.7 | 28 | 2.94 | 1.92~3.91 | 40 | 0.86 | 0.65~1.07 |
| 41 | Survivors | 244 | 297.0 | 277.6~316 | 128 | 1.77 | 1.59~1.95 | 224 | 0.81 | 0.73~0.88 |
| 41 | Non-survivors | 41 | 165.0 | 119.8~212.2 | 26 | 3.56 | 2.44~4.66 | 38 | 0.93 | 0.69~1.16 |
| 42 | Survivors | 218 | 285.5 | 266.4~305.5 | 123 | 1.77 | 1.61~1.92 | 193 | 0.82 | 0.74~0.91 |
| 42 | Non-survivors | 39 | 159.3 | 108.8~209.2 | 26 | 3.39 | 2.47~4.28 | 34 | 0.90 | 0.7~1.12 |
| 43 | Survivors | 210 | 298.3 | 278.6~317.8 | 104 | 1.74 | 1.59~1.89 | 188 | 0.82 | 0.74~0.91 |
| 43 | Non-survivors | 38 | 141.4 | 99.3~182.6 | 26 | 3.40 | 2.4~4.39 | 34 | 0.92 | 0.71~1.13 |
| 44 | Survivors | 202 | 291.5 | 271.7~311.2 | 122 | 1.60 | 1.48~1.72 | 183 | 0.76 | 0.69~0.83 |
| 44 | Non-survivors | 41 | 148.8 | 103.6~192.3 | 28 | 3.15 | 2.35~3.99 | 38 | 0.87 | 0.7~1.05 |
| 45 | Survivors | 184 | 303.8 | 280.5~324.5 | 102 | 1.80 | 1.62~1.98 | 168 | 0.77 | 0.69~0.84 |
| 45 | Non-survivors | 36 | 136.7 | 88.1~180.4 | 22 | 3.04 | 2.25~3.87 | 32 | 0.95 | 0.74~1.17 |
| 46 | Survivors | 181 | 314.6 | 291.6~339 | 105 | 1.74 | 1.59~1.88 | 166 | 0.77 | 0.7~0.84 |
| 46 | Non-survivors | 36 | 153.8 | 101.9~206.1 | 23 | 3.22 | 2.23~4.16 | 28 | 1.09 | 0.81~1.37 |
| 47 | Survivors | 161 | 301.4 | 276.3~325.3 | 94 | 1.82 | 1.58~2.05 | 144 | 0.80 | 0.71~0.89 |
| 47 | Non-survivors | 30 | 149.1 | 94.2~205 | 19 | 3.44 | 2.3~4.54 | 28 | 0.95 | 0.75~1.15 |
| 48 | Survivors | 168 | 301.5 | 276.7~325.7 | 101 | 1.77 | 1.6~1.94 | 154 | 0.76 | 0.69~0.84 |
| 48 | Non-survivors | 28 | 166.6 | 108~224.7 | 17 | 3.81 | 2.25~5.33 | 23 | 0.96 | 0.73~1.19 |
| 49 | Survivors | 159 | 300.7 | 275.4~325.1 | 86 | 1.67 | 1.47~1.85 | 143 | 0.80 | 0.72~0.88 |
| 49 | Non-survivors | 30 | 202.8 | 133.8~269.5 | 15 | 4.67 | 2.04~7.43 | 26 | 0.88 | 0.66~1.09 |
| 50 | Survivors | 155 | 308.0 | 281.5~334.5 | 80 | 1.78 | 1.55~2 | 141 | 0.78 | 0.7~0.86 |
| 50 | Non-survivors | 27 | 172.2 | 114.8~231.7 | 15 | 3.23 | 1.46~5.06 | 20 | 0.81 | 0.61~1.02 |
| 51 | Survivors | 143 | 305.2 | 278~332.9 | 78 | 1.69 | 1.51~1.86 | 127 | 0.86 | 0.75~0.97 |
| 51 | Non-survivors | 26 | 169.5 | 119.8~220.7 | 14 | 2.36 | 1.67~3.02 | 24 | 0.86 | 0.58~1.14 |
| 52 | Survivors | 141 | 312.0 | 283.3~340.5 | 70 | 1.68 | 1.52~1.84 | 129 | 0.81 | 0.71~0.91 |
| 52 | Non-survivors | 27 | 189.5 | 123.4~255 | 15 | 2.69 | 2.05~3.32 | 20 | 0.73 | 0.51~0.97 |
| 53 | Survivors | 123 | 296.6 | 266.6~325.9 | 69 | 1.93 | 1.6~2.24 | 112 | 0.80 | 0.7~0.89 |
| 53 | Non-survivors | 24 | 162.8 | 115~210.3 | 15 | 2.24 | 1.49~2.97 | 21 | 0.75 | 0.56~0.93 |
| 54 | Survivors | 125 | 295.5 | 265.7~324.7 | 68 | 1.78 | 1.53~2.03 | 112 | 0.83 | 0.72~0.95 |
| 54 | Non-survivors | 25 | 166.8 | 109.5~222.4 | 14 | 2.92 | 2.1~3.71 | 21 | 0.66 | 0.48~0.82 |
| 55 | Survivors | 125 | 286.9 | 259.2~316.1 | 70 | 1.69 | 1.52~1.88 | 112 | 0.83 | 0.7~0.94 |
| 55 | Non-survivors | 25 | 172.7 | 112.5~238.1 | 15 | 4.29 | 2.44~6.21 | 23 | 0.73 | 0.55~0.91 |
| 56 | Survivors | 119 | 303.7 | 274.8~334 | 65 | 1.67 | 1.47~1.86 | 101 | 0.83 | 0.72~0.95 |
| 56 | Non-survivors | 26 | 193.0 | 130.2~254 | 15 | 3.41 | 2.14~4.63 | 21 | 0.71 | 0.49~0.94 |

N, number of measurements; CI, confidence interval

B) TB, PT, WBC in forward manner

| Days | Group | TB | | | PT | | | WBC | | |
| --- | --- | --- | --- | --- | --- | --- | --- | --- | --- | --- |
|  |  | N | mean | 95% CI | N | mean | 95% CI | N | mean | 95% CI |
| 0 | Survivors | 1741 | 0.92 | 0.89~0.94 | 1713 | 12.14 | 12.03~12.26 | 1753 | 18.4 | 18~18.8 |
| 0 | Non-survivors | 486 | 1.34 | 1.26~1.42 | 473 | 13.91 | 13.57~14.24 | 490 | 29.1 | 28~30.2 |
| 1 | Survivors | 1507 | 0.99 | 0.97~1.02 | 1069 | 13.08 | 12.87~13.28 | 1730 | 15.0 | 14.7~15.3 |
| 1 | Non-survivors | 432 | 1.21 | 1.12~1.3 | 342 | 17.32 | 16.25~18.41 | 491 | 21.0 | 20.1~21.9 |
| 2 | Survivors | 976 | 0.99 | 0.96~1.02 | 776 | 13.35 | 13.18~13.52 | 1619 | 10.2 | 10~10.5 |
| 2 | Non-survivors | 325 | 1.21 | 1.1~1.3 | 315 | 17.00 | 15.83~18.14 | 482 | 12.5 | 11.9~13.1 |
| 3 | Survivors | 905 | 1.00 | 0.96~1.04 | 769 | 13.10 | 12.93~13.26 | 1448 | 8.5 | 8.3~8.7 |
| 3 | Non-survivors | 300 | 1.27 | 1.16~1.38 | 303 | 15.02 | 14.55~15.47 | 452 | 10.0 | 9.6~10.5 |
| 4 | Survivors | 851 | 1.02 | 0.97~1.07 | 726 | 13.02 | 12.87~13.15 | 1360 | 8.8 | 8.6~9 |
| 4 | Non-survivors | 295 | 1.25 | 1.11~1.4 | 286 | 14.34 | 14.06~14.62 | 428 | 10.2 | 9.7~10.7 |
| 5 | Survivors | 779 | 1.02 | 0.97~1.08 | 656 | 13.02 | 12.89~13.16 | 1242 | 10.1 | 9.9~10.4 |
| 5 | Non-survivors | 252 | 1.21 | 1.06~1.35 | 256 | 14.39 | 14.11~14.66 | 404 | 12.4 | 11.7~13 |
| 6 | Survivors | 711 | 1.00 | 0.94~1.07 | 589 | 12.96 | 12.84~13.08 | 1147 | 12.8 | 12.4~13.1 |
| 6 | Non-survivors | 225 | 1.27 | 1.09~1.45 | 226 | 14.52 | 14.2~14.85 | 380 | 15.9 | 15.2~16.7 |
| 7 | Survivors | 665 | 1.06 | 0.99~1.13 | 553 | 13.13 | 13~13.26 | 1071 | 15.1 | 14.7~15.5 |
| 7 | Non-survivors | 213 | 1.47 | 1.25~1.7 | 202 | 14.79 | 14.38~15.21 | 362 | 17.5 | 16.7~18.3 |
| 8 | Survivors | 612 | 1.08 | 1~1.15 | 473 | 13.14 | 13.01~13.26 | 1003 | 16.0 | 15.6~16.4 |
| 8 | Non-survivors | 189 | 1.78 | 1.48~2.08 | 192 | 15.19 | 14.55~15.81 | 340 | 17.5 | 16.6~18.4 |
| 9 | Survivors | 566 | 1.01 | 0.93~1.08 | 469 | 13.27 | 13.12~13.41 | 952 | 15.8 | 15.4~16.2 |
| 9 | Non-survivors | 190 | 1.85 | 1.51~2.18 | 175 | 15.53 | 14.73~16.32 | 316 | 16.5 | 15.6~17.5 |
| 10 | Survivors | 536 | 1.02 | 0.95~1.1 | 442 | 13.35 | 13.22~13.48 | 903 | 14.6 | 14.3~15 |
| 10 | Non-survivors | 166 | 1.99 | 1.62~2.35 | 172 | 15.68 | 15.01~16.34 | 290 | 15.1 | 14.2~16 |
| 11 | Survivors | 546 | 0.96 | 0.88~1.03 | 428 | 13.42 | 13.28~13.57 | 873 | 13.4 | 13~13.7 |
| 11 | Non-survivors | 154 | 2.15 | 1.7~2.61 | 140 | 15.95 | 15.16~16.74 | 265 | 14.8 | 13.8~15.8 |
| 12 | Survivors | 497 | 0.96 | 0.86~1.06 | 406 | 13.46 | 13.31~13.61 | 831 | 12.6 | 12.2~12.9 |
| 12 | Non-survivors | 126 | 2.12 | 1.6~2.63 | 111 | 15.40 | 14.73~16.13 | 233 | 13.7 | 12.9~14.6 |
| 13 | Survivors | 499 | 0.93 | 0.83~1.02 | 390 | 13.43 | 13.27~13.58 | 796 | 12.1 | 11.7~12.4 |
| 13 | Non-survivors | 133 | 1.75 | 1.43~2.07 | 110 | 15.53 | 14.79~16.25 | 219 | 13.0 | 12.2~13.7 |
| 14 | Survivors | 483 | 0.92 | 0.81~1.02 | 383 | 13.62 | 13.43~13.8 | 761 | 11.8 | 11.5~12.2 |
| 14 | Non-survivors | 122 | 1.91 | 1.56~2.25 | 104 | 15.83 | 15.13~16.49 | 201 | 13.1 | 12.3~14 |
| 15 | Survivors | 460 | 0.86 | 0.77~0.95 | 383 | 13.76 | 13.59~13.93 | 740 | 11.9 | 11.5~12.2 |
| 15 | Non-survivors | 114 | 2.02 | 1.7~2.36 | 103 | 15.81 | 15.02~16.56 | 193 | 13.5 | 12.6~14.3 |
| 16 | Survivors | 432 | 0.86 | 0.77~0.96 | 380 | 13.80 | 13.62~13.98 | 699 | 11.7 | 11.3~12.1 |
| 16 | Non-survivors | 100 | 1.79 | 1.42~2.15 | 92 | 15.58 | 15.03~16.11 | 179 | 13.6 | 12.7~14.4 |
| 17 | Survivors | 417 | 0.86 | 0.77~0.95 | 362 | 13.73 | 13.54~13.92 | 700 | 11.7 | 11.3~12.2 |
| 17 | Non-survivors | 103 | 1.76 | 1.48~2.05 | 88 | 16.05 | 15.22~16.91 | 168 | 14.0 | 13.2~14.9 |
| 18 | Survivors | 415 | 0.82 | 0.74~0.9 | 359 | 13.52 | 13.34~13.69 | 670 | 11.6 | 11.2~12 |
| 18 | Non-survivors | 93 | 1.85 | 1.52~2.2 | 89 | 15.37 | 14.88~15.87 | 160 | 14.8 | 13.6~16 |
| 19 | Survivors | 388 | 0.82 | 0.74~0.91 | 330 | 13.57 | 13.38~13.75 | 637 | 11.5 | 11.1~11.9 |
| 19 | Non-survivors | 91 | 1.90 | 1.5~2.3 | 77 | 15.40 | 14.89~15.94 | 148 | 14.9 | 13.7~16.2 |
| 20 | Survivors | 398 | 0.83 | 0.74~0.91 | 337 | 13.77 | 13.55~13.98 | 630 | 11.7 | 11.3~12.2 |
| 20 | Non-survivors | 99 | 2.27 | 1.73~2.82 | 86 | 16.34 | 15.59~17.04 | 144 | 16.2 | 14.9~17.5 |
| 21 | Survivors | 377 | 0.84 | 0.77~0.92 | 315 | 13.84 | 13.62~14.05 | 592 | 11.8 | 11.3~12.3 |
| 21 | Non-survivors | 88 | 2.10 | 1.65~2.55 | 81 | 16.29 | 15.7~16.89 | 137 | 15.4 | 14.1~16.8 |
| 22 | Survivors | 348 | 0.76 | 0.69~0.83 | 288 | 13.58 | 13.38~13.79 | 577 | 11.6 | 11.1~12.1 |
| 22 | Non-survivors | 82 | 2.35 | 1.77~2.93 | 82 | 18.15 | 15.21~20.97 | 128 | 16.3 | 14.9~17.9 |
| 23 | Survivors | 322 | 0.81 | 0.71~0.9 | 265 | 13.62 | 13.31~13.93 | 538 | 11.6 | 11.1~12.2 |
| 23 | Non-survivors | 73 | 2.71 | 1.87~3.54 | 73 | 16.59 | 15.85~17.33 | 124 | 16.7 | 14.8~18.5 |
| 24 | Survivors | 302 | 0.83 | 0.73~0.93 | 257 | 13.69 | 13.33~14.06 | 515 | 11.4 | 11~11.9 |
| 24 | Non-survivors | 76 | 2.36 | 1.75~2.98 | 71 | 17.45 | 16.43~18.49 | 113 | 17.1 | 14.9~19.2 |
| 25 | Survivors | 309 | 0.84 | 0.74~0.94 | 255 | 13.88 | 13.49~14.28 | 483 | 11.5 | 11~12 |
| 25 | Non-survivors | 61 | 2.60 | 1.77~3.39 | 58 | 16.53 | 15.7~17.35 | 101 | 17.0 | 14.8~19.3 |
| 26 | Survivors | 274 | 0.87 | 0.73~1 | 241 | 13.75 | 13.24~14.24 | 470 | 11.6 | 11.1~12.1 |
| 26 | Non-survivors | 57 | 2.82 | 1.99~3.68 | 56 | 18.70 | 14.05~23.38 | 97 | 17.0 | 14.6~19.2 |
| 27 | Survivors | 276 | 0.86 | 0.74~0.99 | 243 | 13.63 | 13.38~13.88 | 464 | 11.5 | 11~12 |
| 27 | Non-survivors | 55 | 2.75 | 1.95~3.51 | 50 | 16.49 | 15.59~17.33 | 91 | 14.7 | 12.9~16.3 |
| 28 | Survivors | 281 | 0.90 | 0.78~1.02 | 233 | 13.51 | 13.26~13.78 | 445 | 11.0 | 10.5~11.4 |
| 28 | Non-survivors | 56 | 2.58 | 1.73~3.4 | 46 | 17.59 | 16.3~18.81 | 84 | 17.1 | 14.6~19.8 |
| 29 | Survivors | 256 | 0.85 | 0.73~0.97 | 215 | 13.83 | 13.51~14.16 | 415 | 11.1 | 10.7~11.6 |
| 29 | Non-survivors | 48 | 3.10 | 2.01~4.21 | 45 | 16.56 | 15.65~17.43 | 80 | 17.3 | 14.4~20 |
| 30 | Survivors | 269 | 0.86 | 0.74~0.97 | 219 | 13.77 | 13.45~14.09 | 404 | 10.9 | 10.4~11.5 |
| 30 | Non-survivors | 51 | 2.85 | 1.62~3.97 | 47 | 16.89 | 15.88~17.89 | 75 | 18.0 | 15.1~21 |
| 31 | Survivors | 240 | 0.84 | 0.71~0.98 | 207 | 13.80 | 13.47~14.12 | 379 | 10.8 | 10.3~11.2 |
| 31 | Non-survivors | 49 | 3.23 | 2.09~4.42 | 44 | 16.57 | 15.43~17.68 | 73 | 17.8 | 14.8~20.8 |
| 32 | Survivors | 236 | 0.88 | 0.73~1.03 | 202 | 14.03 | 13.62~14.43 | 364 | 10.7 | 10.1~11.2 |
| 32 | Non-survivors | 44 | 3.10 | 1.78~4.43 | 40 | 17.00 | 15.28~18.62 | 70 | 17.3 | 14.4~20.4 |
| 33 | Survivors | 213 | 0.87 | 0.71~1.03 | 175 | 13.73 | 13.4~14.06 | 353 | 10.4 | 9.9~10.9 |
| 33 | Non-survivors | 40 | 3.28 | 1.92~4.6 | 39 | 17.70 | 16.17~19.26 | 67 | 16.0 | 13.2~18.8 |
| 34 | Survivors | 206 | 0.91 | 0.7~1.09 | 191 | 13.58 | 13.27~13.89 | 329 | 10.8 | 10.2~11.4 |
| 34 | Non-survivors | 36 | 3.30 | 1.85~4.75 | 35 | 17.05 | 15.46~18.69 | 59 | 15.9 | 13.2~18.5 |
| 35 | Survivors | 194 | 0.92 | 0.74~1.11 | 179 | 13.66 | 13.33~13.97 | 330 | 10.7 | 10.1~11.3 |
| 35 | Non-survivors | 38 | 2.76 | 1.5~4.03 | 32 | 16.89 | 15.22~18.59 | 56 | 15.4 | 13.2~17.6 |
| 36 | Survivors | 174 | 0.95 | 0.73~1.17 | 138 | 13.57 | 13.22~13.95 | 297 | 10.6 | 10~11.3 |
| 36 | Non-survivors | 36 | 3.40 | 1.91~4.89 | 33 | 17.14 | 15.42~18.95 | 54 | 15.5 | 13.1~17.7 |
| 37 | Survivors | 183 | 0.86 | 0.66~1.08 | 153 | 13.48 | 13.07~13.91 | 285 | 10.3 | 9.6~10.9 |
| 37 | Non-survivors | 34 | 3.42 | 1.85~5.01 | 34 | 16.06 | 14.88~17.25 | 54 | 14.8 | 12.4~17.4 |
| 38 | Survivors | 155 | 0.91 | 0.68~1.13 | 144 | 13.51 | 13.22~13.81 | 254 | 10.4 | 9.8~11.1 |
| 38 | Non-survivors | 32 | 3.14 | 1.71~4.58 | 35 | 20.25 | 12.91~27.88 | 48 | 14.6 | 11.7~17.4 |
| 39 | Survivors | 170 | 0.93 | 0.72~1.14 | 137 | 13.75 | 13.39~14.11 | 264 | 10.3 | 9.7~10.9 |
| 39 | Non-survivors | 25 | 1.94 | 0.81~3 | 29 | 16.12 | 15~17.23 | 43 | 13.5 | 11~15.9 |
| 40 | Survivors | 154 | 1.06 | 0.81~1.31 | 132 | 13.71 | 13.26~14.18 | 246 | 10.2 | 9.6~10.8 |
| 40 | Non-survivors | 27 | 2.08 | 0.97~3.21 | 25 | 17.03 | 14.89~19.2 | 46 | 11.7 | 9.9~13.6 |
| 41 | Survivors | 161 | 1.01 | 0.79~1.24 | 132 | 13.81 | 13.35~14.29 | 244 | 10.1 | 9.5~10.7 |
| 41 | Non-survivors | 30 | 2.08 | 1.13~3.05 | 25 | 16.33 | 14.72~17.88 | 41 | 12.3 | 10.1~14.6 |
| 42 | Survivors | 139 | 1.10 | 0.8~1.38 | 117 | 13.75 | 13.29~14.18 | 218 | 10.6 | 9.7~11.4 |
| 42 | Non-survivors | 22 | 2.28 | 0.94~3.58 | 22 | 17.21 | 15.45~18.94 | 39 | 12.1 | 9.5~14.7 |
| 43 | Survivors | 132 | 0.77 | 0.67~0.88 | 109 | 13.61 | 13.22~13.99 | 210 | 10.0 | 9.4~10.6 |
| 43 | Non-survivors | 22 | 2.56 | 1.24~3.91 | 24 | 17.18 | 15.68~18.7 | 38 | 13.1 | 9.8~16.2 |
| 44 | Survivors | 138 | 0.78 | 0.68~0.88 | 126 | 13.45 | 13.06~13.83 | 202 | 10.1 | 9.5~10.7 |
| 44 | Non-survivors | 26 | 2.10 | 1.16~3.08 | 23 | 15.89 | 14.41~17.38 | 41 | 11.8 | 8.6~15 |
| 45 | Survivors | 120 | 0.99 | 0.65~1.33 | 101 | 13.34 | 12.97~13.71 | 184 | 10.3 | 9.6~11 |
| 45 | Non-survivors | 22 | 2.76 | 1.68~3.78 | 18 | 16.42 | 15.04~17.74 | 36 | 14.0 | 9.9~18 |
| 46 | Survivors | 126 | 1.03 | 0.68~1.38 | 106 | 13.28 | 12.88~13.66 | 181 | 10.5 | 9.8~11.1 |
| 46 | Non-survivors | 19 | 2.18 | 1.26~3.11 | 18 | 16.15 | 14.98~17.32 | 36 | 12.8 | 8.9~16.8 |
| 47 | Survivors | 107 | 1.08 | 0.63~1.52 | 100 | 13.68 | 13.2~14.14 | 161 | 10.8 | 10~11.5 |
| 47 | Non-survivors | 15 | 2.37 | 0.86~3.75 | 17 | 17.53 | 15.04~19.94 | 30 | 10.4 | 8.3~12.6 |
| 48 | Survivors | 123 | 1.04 | 0.7~1.4 | 97 | 13.60 | 13.17~14.04 | 168 | 10.3 | 9.6~10.9 |
| 48 | Non-survivors | 12 | 3.18 | 1.11~5.23 | 13 | 16.64 | 15~18.28 | 28 | 11.2 | 8.3~14.1 |
| 49 | Survivors | 108 | 1.09 | 0.7~1.5 | 98 | 14.87 | 12.18~17.43 | 159 | 9.8 | 9.2~10.3 |
| 49 | Non-survivors | 18 | 2.28 | 0.82~3.72 | 14 | 18.56 | 15.31~21.83 | 30 | 11.7 | 8.8~14.5 |
| 50 | Survivors | 101 | 1.22 | 0.73~1.7 | 82 | 13.63 | 13.01~14.2 | 155 | 9.9 | 9.2~10.5 |
| 50 | Non-survivors | 11 | 3.14 | 1.03~5.21 | 12 | 15.61 | 14.35~16.94 | 27 | 9.9 | 7.6~12.1 |
| 51 | Survivors | 95 | 1.13 | 0.66~1.6 | 82 | 13.63 | 13.02~14.23 | 143 | 10.0 | 9.2~10.7 |
| 51 | Non-survivors | 13 | 2.84 | 0.54~4.96 | 11 | 14.84 | 14.24~15.45 | 26 | 10.2 | 7.6~12.8 |
| 52 | Survivors | 90 | 1.11 | 0.62~1.6 | 71 | 13.62 | 12.9~14.36 | 141 | 10.2 | 9.5~10.9 |
| 52 | Non-survivors | 14 | 3.13 | 0.82~5.46 | 12 | 16.58 | 15.15~17.95 | 27 | 11.6 | 8.2~14.9 |
| 53 | Survivors | 89 | 1.43 | 0.83~2.04 | 76 | 13.56 | 13~14.11 | 123 | 10.5 | 9.6~11.4 |
| 53 | Non-survivors | 16 | 3.16 | 0.99~5.33 | 11 | 15.42 | 14.29~16.51 | 24 | 12.1 | 8.6~15.5 |
| 54 | Survivors | 74 | 1.18 | 0.69~1.67 | 71 | 13.90 | 13.23~14.58 | 125 | 10.6 | 9.5~11.7 |
| 54 | Non-survivors | 12 | 3.39 | 0.4~6.4 | 10 | 29.01 | 2.54~54.53 | 25 | 12.1 | 9.3~14.8 |
| 55 | Survivors | 88 | 1.08 | 0.61~1.5 | 69 | 13.49 | 12.96~14.02 | 125 | 10.1 | 9.2~11.1 |
| 55 | Non-survivors | 13 | 3.65 | 0.57~6.7 | 12 | 15.50 | 13.66~17.46 | 25 | 11.4 | 9~13.7 |
| 56 | Survivors | 71 | 1.45 | 0.8~2.13 | 66 | 13.39 | 12.86~13.88 | 119 | 9.9 | 9.1~10.8 |
| 56 | Non-survivors | 15 | 3.10 | 0.57~5.64 | 12 | 17.48 | 14.73~20.2 | 26 | 12.4 | 9.4~15.4 |

N, number of measurements; CI, confidence interval; TB, total bilirubin; PT, prothrombin time; WBC, white blood cell

2. Tables for the number of measurements, mean, CI in Backward

A) Platelet, Lactate, Creatinine in Backward

| Days | Group | Platelet | | | Lactate | | | Creatinine | | |
| --- | --- | --- | --- | --- | --- | --- | --- | --- | --- | --- |
|  |  | N | Mean | 95% CI | N | Mean | 95% CI | N | Mean | 95% CI |
| -56 | Survivors | 124 | 252.6 | 233.9~270.7 | 121 | 4.68 | 4.24~5.14 | 123 | 1.09 | 0.89~1.29 |
| -56 | Non-survivors | 26 | 247.7 | 210.3~285.6 | 24 | 4.69 | 3.88~5.51 | 26 | 0.98 | 0.79~1.16 |
| -55 | Survivors | 148 | 160.3 | 149.3~171.1 | 128 | 3.65 | 3.25~4.06 | 147 | 1.10 | 0.89~1.3 |
| -55 | Non-survivors | 27 | 166.6 | 135.5~199.6 | 22 | 3.54 | 2.73~4.38 | 27 | 1.04 | 0.84~1.25 |
| -54 | Survivors | 163 | 124.7 | 114.6~135 | 133 | 2.53 | 2.28~2.79 | 157 | 1.00 | 0.86~1.15 |
| -54 | Non-survivors | 27 | 122.9 | 93.3~151.5 | 18 | 2.26 | 1.86~2.65 | 21 | 1.01 | 0.67~1.33 |
| -53 | Survivors | 172 | 112.3 | 102.2~122.5 | 139 | 2.20 | 1.87~2.53 | 169 | 0.91 | 0.79~1.03 |
| -53 | Non-survivors | 25 | 114.8 | 86~143.1 | 18 | 2.12 | 1.26~2.96 | 23 | 0.90 | 0.61~1.18 |
| -52 | Survivors | 182 | 118.0 | 109.1~127.8 | 145 | 1.89 | 1.66~2.13 | 175 | 0.86 | 0.76~0.97 |
| -52 | Non-survivors | 27 | 124.2 | 94.3~150.6 | 17 | 1.52 | 1.25~1.81 | 24 | 0.88 | 0.56~1.18 |
| -51 | Survivors | 189 | 130.6 | 121.8~139.8 | 136 | 1.91 | 1.68~2.15 | 177 | 0.84 | 0.73~0.95 |
| -51 | Non-survivors | 28 | 151.7 | 119~185 | 17 | 2.35 | 0.89~3.86 | 20 | 0.89 | 0.55~1.25 |
| -50 | Survivors | 200 | 152.8 | 142~163.7 | 149 | 1.82 | 1.59~2.04 | 183 | 0.81 | 0.7~0.92 |
| -50 | Non-survivors | 31 | 174.1 | 140.4~207.9 | 20 | 2.06 | 1.48~2.65 | 27 | 0.84 | 0.65~1.04 |
| -49 | Survivors | 212 | 177.5 | 164.7~190.5 | 150 | 1.70 | 1.56~1.84 | 205 | 0.79 | 0.72~0.86 |
| -49 | Non-survivors | 33 | 197.8 | 162.8~235.3 | 20 | 2.27 | 1.6~2.88 | 25 | 0.96 | 0.68~1.25 |
| -48 | Survivors | 215 | 195.1 | 180.7~209.8 | 146 | 1.81 | 1.64~1.98 | 204 | 0.80 | 0.73~0.87 |
| -48 | Non-survivors | 33 | 204.6 | 164.2~245.7 | 17 | 1.88 | 1.5~2.27 | 25 | 0.89 | 0.67~1.11 |
| -47 | Survivors | 222 | 215.2 | 198.9~231.4 | 153 | 1.88 | 1.71~2.05 | 204 | 0.80 | 0.73~0.87 |
| -47 | Non-survivors | 35 | 207.0 | 163.1~253.4 | 22 | 2.22 | 1.88~2.57 | 30 | 0.91 | 0.69~1.14 |
| -46 | Survivors | 234 | 245.0 | 226.5~263.5 | 168 | 1.90 | 1.74~2.07 | 219 | 0.76 | 0.7~0.83 |
| -46 | Non-survivors | 38 | 222.1 | 175.1~269.6 | 24 | 2.00 | 1.64~2.39 | 33 | 0.95 | 0.7~1.19 |
| -45 | Survivors | 246 | 262.4 | 242.1~282.8 | 168 | 1.81 | 1.64~1.98 | 230 | 0.77 | 0.71~0.82 |
| -45 | Non-survivors | 40 | 220.8 | 177.1~263.6 | 25 | 1.86 | 1.53~2.2 | 33 | 0.96 | 0.59~1.33 |
| -44 | Survivors | 259 | 285.2 | 265.5~306 | 178 | 1.85 | 1.68~2.01 | 243 | 0.76 | 0.7~0.81 |
| -44 | Non-survivors | 42 | 231.9 | 182~281.2 | 28 | 2.04 | 1.5~2.54 | 37 | 0.94 | 0.64~1.24 |
| -43 | Survivors | 272 | 306.6 | 284.6~329.7 | 179 | 1.83 | 1.65~2.01 | 248 | 0.77 | 0.7~0.84 |
| -43 | Non-survivors | 44 | 264.3 | 209.8~319.9 | 31 | 2.18 | 1.78~2.59 | 41 | 0.84 | 0.65~1.02 |
| -42 | Survivors | 284 | 320.3 | 297.2~343.4 | 207 | 1.89 | 1.73~2.06 | 268 | 0.74 | 0.68~0.8 |
| -42 | Non-survivors | 42 | 254.0 | 196.9~316 | 29 | 1.97 | 1.6~2.33 | 41 | 0.78 | 0.63~0.94 |
| -41 | Survivors | 293 | 331.8 | 307.5~355.4 | 191 | 1.86 | 1.69~2.04 | 267 | 0.76 | 0.7~0.81 |
| -41 | Non-survivors | 45 | 260.1 | 208.9~312.9 | 30 | 2.02 | 1.62~2.42 | 41 | 0.77 | 0.63~0.91 |
| -40 | Survivors | 300 | 340.9 | 316.9~366.3 | 208 | 1.99 | 1.79~2.19 | 275 | 0.75 | 0.7~0.8 |
| -40 | Non-survivors | 45 | 256.5 | 206.5~306.5 | 32 | 1.99 | 1.57~2.4 | 42 | 0.99 | 0.52~1.45 |
| -39 | Survivors | 322 | 344.3 | 319.3~367.7 | 211 | 2.03 | 1.84~2.21 | 296 | 0.74 | 0.69~0.79 |
| -39 | Non-survivors | 44 | 237.7 | 192.1~284 | 32 | 2.01 | 1.58~2.44 | 41 | 0.93 | 0.58~1.29 |
| -38 | Survivors | 339 | 342.8 | 318.9~366.6 | 226 | 1.97 | 1.8~2.13 | 307 | 0.75 | 0.7~0.8 |
| -38 | Non-survivors | 50 | 240.6 | 197~284 | 35 | 2.15 | 1.72~2.58 | 45 | 0.98 | 0.7~1.26 |
| -37 | Survivors | 351 | 333.6 | 310.5~356.7 | 221 | 1.96 | 1.76~2.15 | 311 | 0.73 | 0.68~0.77 |
| -37 | Non-survivors | 53 | 224.1 | 190.9~256 | 41 | 2.45 | 1.95~2.95 | 51 | 0.92 | 0.71~1.14 |
| -36 | Survivors | 367 | 321.9 | 299.6~345.1 | 244 | 1.96 | 1.81~2.1 | 330 | 0.73 | 0.69~0.77 |
| -36 | Non-survivors | 56 | 205.4 | 175.2~237.3 | 44 | 2.37 | 1.93~2.8 | 54 | 0.87 | 0.67~1.06 |
| -35 | Survivors | 396 | 315.4 | 295.2~335.9 | 269 | 1.96 | 1.81~2.1 | 370 | 0.74 | 0.7~0.79 |
| -35 | Non-survivors | 59 | 204.5 | 171.6~237.6 | 41 | 2.10 | 1.53~2.69 | 55 | 0.84 | 0.69~0.99 |
| -34 | Survivors | 410 | 303.7 | 284.7~322.5 | 272 | 1.92 | 1.79~2.05 | 371 | 0.75 | 0.7~0.79 |
| -34 | Non-survivors | 61 | 206.4 | 171.9~241.1 | 43 | 2.13 | 1.61~2.68 | 56 | 0.87 | 0.71~1.03 |
| -33 | Survivors | 421 | 297.8 | 278.7~316.2 | 262 | 1.94 | 1.81~2.06 | 393 | 0.75 | 0.7~0.79 |
| -33 | Non-survivors | 66 | 216.4 | 175.5~259.2 | 52 | 2.54 | 2.06~3.03 | 62 | 0.90 | 0.73~1.09 |
| -32 | Survivors | 453 | 296.7 | 279.4~313.5 | 291 | 1.90 | 1.77~2.05 | 421 | 0.75 | 0.71~0.8 |
| -32 | Non-survivors | 67 | 197.4 | 164~230.2 | 50 | 2.46 | 1.97~2.92 | 62 | 0.92 | 0.76~1.07 |
| -31 | Survivors | 461 | 299.6 | 282~317 | 287 | 2.00 | 1.83~2.17 | 422 | 0.74 | 0.7~0.78 |
| -31 | Non-survivors | 76 | 193.1 | 160.8~224.7 | 54 | 2.84 | 2.31~3.37 | 66 | 0.94 | 0.79~1.1 |
| -30 | Survivors | 476 | 300.8 | 284.4~317.8 | 283 | 1.84 | 1.71~1.97 | 422 | 0.73 | 0.69~0.77 |
| -30 | Non-survivors | 76 | 191.9 | 157~226.1 | 54 | 2.18 | 1.85~2.5 | 69 | 0.92 | 0.76~1.08 |
| -29 | Survivors | 481 | 303.8 | 286.2~320.6 | 298 | 1.87 | 1.73~2 | 431 | 0.75 | 0.71~0.8 |
| -29 | Non-survivors | 82 | 190.6 | 160.9~222.5 | 55 | 2.21 | 1.9~2.5 | 73 | 0.93 | 0.74~1.11 |
| -28 | Survivors | 513 | 306.1 | 289.2~322.4 | 327 | 1.89 | 1.76~2.03 | 482 | 0.74 | 0.69~0.78 |
| -28 | Non-survivors | 86 | 177.2 | 150.3~203.7 | 58 | 2.43 | 1.97~2.89 | 80 | 0.95 | 0.82~1.09 |
| -27 | Survivors | 516 | 311.1 | 295.3~327 | 300 | 1.84 | 1.72~1.97 | 467 | 0.75 | 0.7~0.79 |
| -27 | Non-survivors | 92 | 185.0 | 156.1~214.8 | 57 | 2.24 | 1.86~2.62 | 86 | 0.93 | 0.8~1.06 |
| -26 | Survivors | 535 | 312.5 | 295.9~329.2 | 314 | 1.86 | 1.75~1.98 | 481 | 0.76 | 0.72~0.8 |
| -26 | Non-survivors | 99 | 172.2 | 146.6~198.7 | 65 | 2.70 | 2.19~3.2 | 93 | 0.93 | 0.81~1.07 |
| -25 | Survivors | 558 | 320.4 | 304.7~336.8 | 348 | 1.90 | 1.79~2.01 | 511 | 0.79 | 0.73~0.85 |
| -25 | Non-survivors | 104 | 171.4 | 147.9~196 | 71 | 2.17 | 1.91~2.44 | 95 | 0.91 | 0.79~1.03 |
| -24 | Survivors | 566 | 320.8 | 304.8~337.4 | 335 | 1.92 | 1.76~2.07 | 520 | 0.79 | 0.74~0.85 |
| -24 | Non-survivors | 116 | 171.9 | 148.8~196.1 | 78 | 2.51 | 2.12~2.9 | 109 | 0.92 | 0.8~1.04 |
| -23 | Survivors | 582 | 322.6 | 306.1~339.6 | 329 | 1.89 | 1.77~2.02 | 518 | 0.78 | 0.73~0.82 |
| -23 | Non-survivors | 125 | 167.6 | 147.2~188.8 | 84 | 2.60 | 2.16~3.03 | 117 | 0.87 | 0.77~0.96 |
| -22 | Survivors | 607 | 322.8 | 305.8~339.1 | 345 | 1.83 | 1.73~1.94 | 544 | 0.78 | 0.73~0.82 |
| -22 | Non-survivors | 130 | 165.4 | 147.8~184.4 | 84 | 2.20 | 1.98~2.44 | 118 | 0.87 | 0.78~0.97 |
| -21 | Survivors | 647 | 325.1 | 309.5~340.6 | 410 | 1.84 | 1.74~1.94 | 596 | 0.76 | 0.72~0.8 |
| -21 | Non-survivors | 138 | 170.4 | 149.7~191.6 | 90 | 2.32 | 1.97~2.66 | 128 | 0.90 | 0.79~1 |
| -20 | Survivors | 648 | 323.3 | 307.3~338.8 | 360 | 1.97 | 1.84~2.1 | 570 | 0.77 | 0.72~0.81 |
| -20 | Non-survivors | 145 | 171.7 | 151.4~192.1 | 100 | 2.35 | 2.09~2.6 | 133 | 0.92 | 0.81~1.03 |
| -19 | Survivors | 677 | 324.7 | 309.1~340.6 | 372 | 1.91 | 1.79~2.03 | 591 | 0.75 | 0.71~0.79 |
| -19 | Non-survivors | 151 | 172.9 | 153.7~192.3 | 103 | 2.44 | 2.15~2.75 | 143 | 0.93 | 0.83~1.03 |
| -18 | Survivors | 666 | 325.6 | 310.1~341.6 | 404 | 1.84 | 1.73~1.95 | 607 | 0.76 | 0.72~0.81 |
| -18 | Non-survivors | 163 | 175.4 | 157.2~194.3 | 117 | 2.41 | 2.13~2.7 | 152 | 0.94 | 0.84~1.06 |
| -17 | Survivors | 724 | 325.4 | 311.1~339.5 | 407 | 1.83 | 1.73~1.93 | 656 | 0.75 | 0.71~0.79 |
| -17 | Non-survivors | 170 | 175.7 | 156.5~194.2 | 113 | 2.47 | 2.2~2.77 | 156 | 0.96 | 0.86~1.08 |
| -16 | Survivors | 718 | 324.0 | 309.6~338.6 | 392 | 1.81 | 1.71~1.91 | 643 | 0.76 | 0.71~0.8 |
| -16 | Non-survivors | 183 | 175.3 | 156.2~194.3 | 119 | 2.55 | 2.27~2.82 | 171 | 0.95 | 0.85~1.05 |
| -15 | Survivors | 714 | 318.4 | 305~331.6 | 400 | 1.76 | 1.68~1.85 | 637 | 0.75 | 0.71~0.79 |
| -15 | Non-survivors | 194 | 173.0 | 155.2~191 | 129 | 3.01 | 2.58~3.41 | 178 | 1.00 | 0.89~1.1 |
| -14 | Survivors | 792 | 328.1 | 315.5~341.3 | 483 | 1.77 | 1.69~1.86 | 739 | 0.74 | 0.7~0.78 |
| -14 | Non-survivors | 202 | 167.3 | 150.7~184.8 | 131 | 2.73 | 2.38~3.08 | 185 | 0.98 | 0.88~1.08 |
| -13 | Survivors | 762 | 326.3 | 313~339.5 | 406 | 1.81 | 1.7~1.92 | 674 | 0.75 | 0.71~0.78 |
| -13 | Non-survivors | 215 | 163.8 | 147.4~181.3 | 142 | 2.68 | 2.34~2.99 | 194 | 1.00 | 0.9~1.09 |
| -12 | Survivors | 795 | 327.0 | 314.8~339.9 | 424 | 1.78 | 1.69~1.87 | 698 | 0.74 | 0.7~0.77 |
| -12 | Non-survivors | 237 | 165.2 | 149.2~180.7 | 161 | 2.94 | 2.52~3.36 | 215 | 1.00 | 0.91~1.09 |
| -11 | Survivors | 828 | 330.7 | 318.5~342.8 | 486 | 1.87 | 1.76~1.98 | 744 | 0.74 | 0.7~0.78 |
| -11 | Non-survivors | 265 | 155.9 | 140.9~170.5 | 183 | 2.90 | 2.59~3.21 | 239 | 1.06 | 0.96~1.17 |
| -10 | Survivors | 857 | 329.7 | 318~342.3 | 460 | 1.73 | 1.64~1.82 | 771 | 0.74 | 0.7~0.77 |
| -10 | Non-survivors | 292 | 154.7 | 139.7~168.7 | 200 | 3.17 | 2.85~3.51 | 271 | 1.06 | 0.98~1.15 |
| -9 | Survivors | 853 | 331.6 | 319.7~343.1 | 450 | 1.70 | 1.61~1.79 | 765 | 0.73 | 0.69~0.77 |
| -9 | Non-survivors | 308 | 144.9 | 132.1~157.2 | 218 | 3.08 | 2.8~3.36 | 289 | 1.07 | 0.98~1.17 |
| -8 | Survivors | 869 | 325.1 | 313.5~336.6 | 471 | 1.84 | 1.71~1.97 | 794 | 0.72 | 0.68~0.76 |
| -8 | Non-survivors | 337 | 142.9 | 131.4~154.4 | 236 | 3.08 | 2.78~3.39 | 308 | 1.07 | 0.98~1.16 |
| -7 | Survivors | 1019 | 330.5 | 319.4~341.6 | 587 | 1.86 | 1.77~1.96 | 951 | 0.72 | 0.68~0.75 |
| -7 | Non-survivors | 361 | 143.3 | 131.4~155.9 | 249 | 3.18 | 2.85~3.53 | 342 | 1.07 | 0.99~1.16 |
| -6 | Survivors | 986 | 321.2 | 309.7~332.3 | 518 | 1.82 | 1.73~1.93 | 877 | 0.74 | 0.7~0.78 |
| -6 | Non-survivors | 370 | 131.4 | 119.9~142.6 | 258 | 2.96 | 2.7~3.23 | 352 | 1.06 | 0.98~1.14 |
| -5 | Survivors | 1042 | 315.6 | 304.8~325.9 | 558 | 1.81 | 1.71~1.91 | 941 | 0.73 | 0.7~0.76 |
| -5 | Non-survivors | 398 | 129.1 | 118.5~139.4 | 279 | 3.07 | 2.83~3.32 | 380 | 1.08 | 1~1.15 |
| -4 | Survivors | 1173 | 312.1 | 302.7~321.8 | 653 | 1.80 | 1.71~1.88 | 1080 | 0.72 | 0.69~0.75 |
| -4 | Non-survivors | 428 | 120.9 | 111.1~130.7 | 308 | 3.34 | 3.07~3.6 | 401 | 1.14 | 1.06~1.21 |
| -3 | Survivors | 1237 | 313.2 | 303.2~322.5 | 644 | 1.77 | 1.67~1.87 | 1132 | 0.70 | 0.67~0.73 |
| -3 | Non-survivors | 442 | 105.7 | 97.1~114.6 | 303 | 3.28 | 3.02~3.55 | 422 | 1.25 | 1.16~1.34 |
| -2 | Survivors | 1293 | 305.3 | 296.2~314.3 | 676 | 1.73 | 1.64~1.8 | 1163 | 0.71 | 0.68~0.73 |
| -2 | Non-survivors | 467 | 105.7 | 94.8~115.4 | 325 | 3.70 | 3.43~3.99 | 448 | 1.37 | 1.27~1.47 |
| -1 | Survivors | 1200 | 302.6 | 293.6~312.1 | 624 | 1.64 | 1.55~1.72 | 1090 | 0.71 | 0.68~0.74 |
| -1 | Non-survivors | 478 | 83.2 | 75.4~90.9 | 329 | 4.28 | 3.89~4.68 | 461 | 1.56 | 1.46~1.68 |
| 0 | Survivors | 1658 | 337.5 | 329.3~345.9 | 692 | 1.55 | 1.49~1.6 | 1499 | 0.69 | 0.66~0.71 |
| 0 | Non-survivors | 470 | 76.5 | 68.5~84.4 | 303 | 5.25 | 4.75~5.74 | 432 | 1.85 | 1.73~1.99 |

N, number of measurements; CI, confidence interval

B) TB, PT, WBC in Backward

| Days | Group | TB | | | PT | | | WBC | | |
| --- | --- | --- | --- | --- | --- | --- | --- | --- | --- | --- |
|  |  | N | Mean | 95% CI | N | Mean | 95% CI | N | Mean | 95% CI |
| -56 | Survivors | 123 | 1.20 | 1.08~1.3 | 122 | 12.06 | 11.77~12.35 | 124 | 26.2 | 24.4~28 |
| -56 | Non-survivors | 26 | 0.93 | 0.72~1.12 | 26 | 12.24 | 11.66~12.81 | 26 | 22.1 | 18~26.1 |
| -55 | Survivors | 137 | 1.06 | 0.94~1.18 | 120 | 13.42 | 12.97~13.86 | 148 | 20.7 | 19.2~22.1 |
| -55 | Non-survivors | 24 | 0.75 | 0.62~0.89 | 14 | 13.13 | 11.96~14.29 | 27 | 17.2 | 14~20.7 |
| -54 | Survivors | 123 | 1.05 | 0.94~1.17 | 118 | 13.60 | 13.1~14.1 | 163 | 14.7 | 13.3~16 |
| -54 | Non-survivors | 17 | 0.87 | 0.62~1.12 | 8 | 12.35 | 11.25~13.4 | 27 | 11.1 | 9.1~13.2 |
| -53 | Survivors | 121 | 1.11 | 0.99~1.22 | 121 | 13.49 | 13.01~13.97 | 172 | 11.9 | 10.8~13 |
| -53 | Non-survivors | 16 | 0.91 | 0.67~1.16 | 12 | 13.96 | 12.58~15.36 | 25 | 9.5 | 8~11 |
| -52 | Survivors | 133 | 1.23 | 1.08~1.37 | 136 | 13.25 | 12.96~13.53 | 182 | 11.2 | 10.4~12.2 |
| -52 | Non-survivors | 19 | 0.84 | 0.64~1.04 | 15 | 12.59 | 11.9~13.3 | 27 | 9.5 | 7.9~11.1 |
| -51 | Survivors | 132 | 1.27 | 1.09~1.46 | 128 | 13.19 | 12.87~13.49 | 189 | 12.4 | 11.4~13.2 |
| -51 | Non-survivors | 15 | 0.99 | 0.67~1.3 | 15 | 13.11 | 12.3~13.94 | 28 | 12.0 | 9.9~14 |
| -50 | Survivors | 138 | 1.21 | 1.04~1.37 | 130 | 12.99 | 12.75~13.23 | 200 | 14.4 | 13.4~15.4 |
| -50 | Non-survivors | 20 | 1.13 | 0.82~1.42 | 16 | 13.62 | 12.72~14.5 | 31 | 14.7 | 12.4~17.2 |
| -49 | Survivors | 169 | 1.26 | 1.11~1.42 | 151 | 13.29 | 13.04~13.55 | 212 | 16.6 | 15.6~17.6 |
| -49 | Non-survivors | 17 | 1.08 | 0.7~1.45 | 16 | 13.81 | 12.84~14.76 | 33 | 17.3 | 15~19.5 |
| -48 | Survivors | 152 | 1.31 | 1.1~1.52 | 145 | 13.33 | 13.06~13.6 | 215 | 16.6 | 15.6~17.5 |
| -48 | Non-survivors | 17 | 1.46 | 0.96~1.98 | 15 | 13.96 | 12.47~15.42 | 33 | 15.7 | 13.5~17.8 |
| -47 | Survivors | 151 | 1.24 | 1.07~1.42 | 140 | 13.74 | 13.09~14.36 | 222 | 16.2 | 15.3~17.1 |
| -47 | Non-survivors | 25 | 1.28 | 0.95~1.61 | 22 | 14.28 | 13.34~15.26 | 35 | 15.8 | 13.4~18.1 |
| -46 | Survivors | 165 | 1.16 | 0.99~1.33 | 161 | 13.47 | 13.16~13.81 | 234 | 16.4 | 15.4~17.3 |
| -46 | Non-survivors | 25 | 1.37 | 1~1.75 | 18 | 14.33 | 12.88~15.67 | 38 | 15.3 | 13.1~17.5 |
| -45 | Survivors | 178 | 1.19 | 1~1.38 | 162 | 13.54 | 13.29~13.79 | 246 | 15.1 | 14.2~16 |
| -45 | Non-survivors | 26 | 1.30 | 0.98~1.63 | 26 | 14.35 | 13.5~15.24 | 40 | 15.0 | 12.9~17.3 |
| -44 | Survivors | 173 | 1.26 | 1.02~1.5 | 171 | 13.55 | 13.22~13.86 | 259 | 14.6 | 13.8~15.4 |
| -44 | Non-survivors | 26 | 1.31 | 0.93~1.68 | 25 | 14.21 | 12.87~15.52 | 42 | 15.1 | 13~17.2 |
| -43 | Survivors | 178 | 1.22 | 0.98~1.46 | 166 | 13.50 | 13.06~13.94 | 272 | 13.8 | 13.1~14.5 |
| -43 | Non-survivors | 33 | 1.27 | 1~1.56 | 27 | 14.99 | 13.26~16.77 | 44 | 14.4 | 12.5~16.2 |
| -42 | Survivors | 217 | 1.12 | 0.95~1.29 | 194 | 13.41 | 13.16~13.65 | 284 | 13.4 | 12.7~14.1 |
| -42 | Non-survivors | 32 | 1.21 | 0.93~1.5 | 26 | 14.71 | 13.87~15.55 | 42 | 14.3 | 12.8~15.8 |
| -41 | Survivors | 186 | 1.12 | 0.94~1.29 | 190 | 13.68 | 13.41~13.94 | 293 | 13.1 | 12.5~13.9 |
| -41 | Non-survivors | 30 | 1.23 | 0.98~1.48 | 25 | 13.52 | 12.93~14.09 | 45 | 13.7 | 11.9~15.5 |
| -40 | Survivors | 202 | 1.08 | 0.93~1.22 | 191 | 13.75 | 13.48~14.01 | 300 | 12.9 | 12.2~13.5 |
| -40 | Non-survivors | 35 | 1.19 | 0.9~1.46 | 29 | 14.65 | 12.94~16.27 | 45 | 14.0 | 12~16 |
| -39 | Survivors | 219 | 1.03 | 0.9~1.18 | 202 | 13.59 | 13.34~13.84 | 322 | 13.1 | 12.5~13.8 |
| -39 | Non-survivors | 31 | 1.30 | 0.95~1.67 | 26 | 13.98 | 13.28~14.63 | 44 | 15.4 | 13.1~17.7 |
| -38 | Survivors | 223 | 0.98 | 0.87~1.08 | 210 | 13.71 | 13.42~14 | 339 | 13.7 | 12.9~14.5 |
| -38 | Non-survivors | 38 | 1.18 | 0.88~1.47 | 34 | 15.31 | 13.98~16.62 | 50 | 15.0 | 12.8~17.2 |
| -37 | Survivors | 217 | 1.03 | 0.91~1.16 | 209 | 13.56 | 13.27~13.84 | 351 | 13.4 | 12.7~14.1 |
| -37 | Non-survivors | 44 | 1.31 | 0.97~1.66 | 34 | 14.88 | 13.34~16.35 | 53 | 16.7 | 14.1~19.2 |
| -36 | Survivors | 241 | 1.10 | 0.97~1.22 | 235 | 13.62 | 13.31~13.92 | 367 | 13.9 | 13.3~14.6 |
| -36 | Non-survivors | 43 | 1.47 | 0.96~1.94 | 41 | 15.84 | 13.72~17.95 | 56 | 16.8 | 14.4~19.3 |
| -35 | Survivors | 298 | 0.99 | 0.9~1.07 | 267 | 13.64 | 13.35~13.92 | 396 | 13.4 | 12.8~14 |
| -35 | Non-survivors | 41 | 1.65 | 0.95~2.32 | 38 | 15.95 | 14.34~17.59 | 59 | 13.7 | 12~15.3 |
| -34 | Survivors | 254 | 1.02 | 0.91~1.12 | 254 | 13.56 | 13.31~13.8 | 410 | 13.5 | 12.8~14.2 |
| -34 | Non-survivors | 42 | 1.58 | 1~2.14 | 40 | 16.21 | 14.09~18.4 | 61 | 13.3 | 11.7~15 |
| -33 | Survivors | 275 | 1.05 | 0.94~1.16 | 253 | 13.70 | 13.39~14.03 | 421 | 13.2 | 12.6~13.8 |
| -33 | Non-survivors | 49 | 1.53 | 1.02~2.07 | 45 | 14.91 | 13.95~15.81 | 66 | 15.1 | 13~17.3 |
| -32 | Survivors | 304 | 1.01 | 0.91~1.1 | 283 | 13.63 | 13.31~13.94 | 453 | 13.2 | 12.6~13.8 |
| -32 | Non-survivors | 50 | 1.54 | 0.94~2.14 | 43 | 14.87 | 14.17~15.57 | 67 | 15.8 | 13.6~17.8 |
| -31 | Survivors | 312 | 0.97 | 0.87~1.07 | 281 | 13.57 | 13.23~13.91 | 461 | 13.6 | 12.9~14.3 |
| -31 | Non-survivors | 53 | 1.59 | 0.97~2.21 | 46 | 15.12 | 14.39~15.79 | 76 | 15.1 | 13.4~17 |
| -30 | Survivors | 289 | 1.01 | 0.87~1.14 | 276 | 13.61 | 13.21~13.98 | 476 | 13.1 | 12.5~13.7 |
| -30 | Non-survivors | 52 | 1.51 | 0.96~2.04 | 50 | 15.41 | 14.58~16.31 | 76 | 14.0 | 12.5~15.5 |
| -29 | Survivors | 318 | 1.06 | 0.93~1.19 | 292 | 13.59 | 13.18~14.04 | 481 | 13.0 | 12.4~13.6 |
| -29 | Non-survivors | 52 | 1.49 | 1.05~1.94 | 48 | 15.00 | 14.13~15.87 | 82 | 13.7 | 12.3~15.2 |
| -28 | Survivors | 390 | 0.98 | 0.88~1.07 | 309 | 13.42 | 13.21~13.63 | 513 | 12.8 | 12.2~13.3 |
| -28 | Non-survivors | 61 | 1.37 | 1.03~1.71 | 51 | 14.26 | 13.69~14.8 | 86 | 14.6 | 13~16.2 |
| -27 | Survivors | 306 | 0.98 | 0.88~1.09 | 289 | 13.51 | 13.29~13.73 | 516 | 12.6 | 12.1~13.1 |
| -27 | Non-survivors | 53 | 1.55 | 1.16~1.95 | 45 | 14.46 | 13.88~15.07 | 92 | 14.1 | 12.7~15.5 |
| -26 | Survivors | 326 | 1.01 | 0.9~1.12 | 291 | 13.39 | 13.16~13.64 | 535 | 12.5 | 12~13 |
| -26 | Non-survivors | 66 | 1.31 | 1.04~1.57 | 60 | 14.32 | 13.85~14.77 | 99 | 15.4 | 13.7~17.2 |
| -25 | Survivors | 368 | 1.00 | 0.89~1.11 | 318 | 13.48 | 13.24~13.71 | 558 | 12.1 | 11.7~12.6 |
| -25 | Non-survivors | 70 | 1.32 | 1.08~1.57 | 62 | 14.60 | 13.95~15.22 | 104 | 14.2 | 12.9~15.6 |
| -24 | Survivors | 371 | 0.97 | 0.85~1.09 | 314 | 13.64 | 13.38~13.89 | 566 | 12.2 | 11.7~12.6 |
| -24 | Non-survivors | 77 | 1.29 | 1.04~1.55 | 67 | 14.86 | 14.25~15.5 | 116 | 14.0 | 12.7~15.4 |
| -23 | Survivors | 341 | 0.97 | 0.86~1.08 | 323 | 13.56 | 13.32~13.79 | 582 | 11.8 | 11.3~12.3 |
| -23 | Non-survivors | 81 | 1.33 | 1.09~1.57 | 62 | 15.61 | 14.83~16.42 | 125 | 13.8 | 12.4~15.2 |
| -22 | Survivors | 376 | 0.97 | 0.85~1.09 | 338 | 13.49 | 13.29~13.7 | 607 | 11.9 | 11.4~12.4 |
| -22 | Non-survivors | 80 | 1.45 | 1.18~1.71 | 67 | 14.73 | 14.01~15.44 | 130 | 14.1 | 12.9~15.3 |
| -21 | Survivors | 475 | 0.98 | 0.87~1.09 | 399 | 13.59 | 13.39~13.8 | 647 | 12.0 | 11.6~12.5 |
| -21 | Non-survivors | 90 | 1.17 | 0.96~1.37 | 76 | 15.11 | 14.52~15.7 | 138 | 13.7 | 12.5~14.8 |
| -20 | Survivors | 386 | 1.08 | 0.96~1.2 | 343 | 13.58 | 13.31~13.84 | 648 | 12.0 | 11.6~12.5 |
| -20 | Non-survivors | 93 | 1.36 | 1.06~1.62 | 81 | 14.58 | 14.1~15.03 | 145 | 14.0 | 12.8~15.1 |
| -19 | Survivors | 404 | 1.01 | 0.87~1.13 | 330 | 13.36 | 13.15~13.56 | 677 | 11.9 | 11.4~12.4 |
| -19 | Non-survivors | 99 | 1.46 | 1.18~1.72 | 86 | 14.74 | 14.26~15.23 | 151 | 14.4 | 13.1~15.6 |
| -18 | Survivors | 433 | 0.92 | 0.83~1.01 | 365 | 13.58 | 13.38~13.79 | 666 | 11.8 | 11.4~12.2 |
| -18 | Non-survivors | 105 | 1.32 | 1.09~1.55 | 99 | 15.05 | 14.63~15.49 | 163 | 14.0 | 12.9~15.2 |
| -17 | Survivors | 473 | 0.94 | 0.84~1.02 | 385 | 13.45 | 13.24~13.67 | 724 | 11.6 | 11.3~12 |
| -17 | Non-survivors | 105 | 1.30 | 1.05~1.54 | 99 | 15.02 | 14.52~15.55 | 170 | 14.3 | 13.1~15.5 |
| -16 | Survivors | 416 | 1.08 | 0.96~1.19 | 375 | 13.45 | 13.23~13.68 | 718 | 12.0 | 11.5~12.5 |
| -16 | Non-survivors | 112 | 1.43 | 1.19~1.66 | 102 | 15.79 | 13.69~17.86 | 183 | 14.5 | 13.3~15.7 |
| -15 | Survivors | 441 | 0.95 | 0.86~1.04 | 366 | 13.34 | 13.13~13.54 | 714 | 11.5 | 11.1~11.9 |
| -15 | Non-survivors | 125 | 1.42 | 1.18~1.66 | 100 | 14.82 | 14.24~15.34 | 194 | 15.5 | 14.1~16.9 |
| -14 | Survivors | 576 | 0.88 | 0.8~0.97 | 454 | 13.31 | 13.12~13.49 | 792 | 11.8 | 11.4~12.2 |
| -14 | Non-survivors | 121 | 1.37 | 1.15~1.58 | 102 | 15.07 | 14.48~15.68 | 202 | 15.1 | 13.9~16.4 |
| -13 | Survivors | 442 | 0.89 | 0.83~0.96 | 371 | 13.13 | 12.94~13.33 | 762 | 11.6 | 11.2~12 |
| -13 | Non-survivors | 132 | 1.55 | 1.26~1.82 | 118 | 15.07 | 14.55~15.6 | 215 | 14.8 | 13.7~15.9 |
| -12 | Survivors | 461 | 0.86 | 0.8~0.94 | 392 | 13.16 | 12.97~13.34 | 795 | 11.6 | 11.2~12 |
| -12 | Non-survivors | 143 | 1.44 | 1.2~1.67 | 140 | 14.85 | 14.42~15.31 | 237 | 15.3 | 14.2~16.4 |
| -11 | Survivors | 544 | 0.84 | 0.75~0.94 | 437 | 13.28 | 12.93~13.66 | 828 | 11.5 | 11.1~11.8 |
| -11 | Non-survivors | 179 | 1.59 | 1.35~1.83 | 161 | 15.61 | 14.74~16.48 | 265 | 16.1 | 14.8~17.4 |
| -10 | Survivors | 542 | 0.86 | 0.77~0.96 | 439 | 13.13 | 12.93~13.33 | 857 | 11.5 | 11.1~11.9 |
| -10 | Non-survivors | 192 | 1.66 | 1.38~1.93 | 183 | 15.68 | 14.15~17.22 | 292 | 15.6 | 14.6~16.6 |
| -9 | Survivors | 522 | 0.88 | 0.78~0.97 | 427 | 13.18 | 12.99~13.38 | 853 | 11.4 | 11~11.7 |
| -9 | Non-survivors | 193 | 1.54 | 1.29~1.78 | 200 | 15.35 | 14.9~15.82 | 308 | 16.0 | 15~17.1 |
| -8 | Survivors | 544 | 0.87 | 0.77~0.96 | 435 | 13.38 | 13.09~13.68 | 869 | 11.3 | 11~11.7 |
| -8 | Non-survivors | 210 | 1.70 | 1.4~1.98 | 217 | 15.33 | 14.88~15.8 | 337 | 15.2 | 14.2~16.1 |
| -7 | Survivors | 756 | 0.83 | 0.75~0.9 | 598 | 13.36 | 12.93~13.8 | 1019 | 11.6 | 11.2~11.9 |
| -7 | Non-survivors | 238 | 1.80 | 1.52~2.09 | 236 | 15.41 | 14.95~15.89 | 361 | 15.9 | 14.8~17 |
| -6 | Survivors | 578 | 0.93 | 0.83~1.02 | 476 | 13.06 | 12.88~13.23 | 986 | 11.7 | 11.3~12.1 |
| -6 | Non-survivors | 244 | 1.84 | 1.54~2.12 | 243 | 15.51 | 15.07~15.96 | 370 | 15.4 | 14.5~16.5 |
| -5 | Survivors | 664 | 0.87 | 0.8~0.95 | 518 | 13.06 | 12.88~13.23 | 1042 | 11.4 | 11~11.7 |
| -5 | Non-survivors | 264 | 1.99 | 1.67~2.3 | 267 | 15.61 | 15.18~16.07 | 398 | 15.8 | 14.8~16.7 |
| -4 | Survivors | 824 | 0.84 | 0.77~0.91 | 611 | 13.00 | 12.83~13.17 | 1173 | 11.3 | 11~11.6 |
| -4 | Non-survivors | 293 | 2.11 | 1.79~2.47 | 280 | 15.59 | 15.21~15.97 | 428 | 16.4 | 15.5~17.3 |
| -3 | Survivors | 826 | 0.82 | 0.75~0.9 | 615 | 12.87 | 12.72~13.01 | 1237 | 11.1 | 10.9~11.4 |
| -3 | Non-survivors | 292 | 2.34 | 1.97~2.7 | 277 | 16.27 | 15.79~16.78 | 442 | 16.6 | 15.6~17.5 |
| -2 | Survivors | 806 | 0.82 | 0.77~0.88 | 639 | 12.85 | 12.69~13.02 | 1293 | 11.1 | 10.8~11.3 |
| -2 | Non-survivors | 298 | 2.56 | 2.2~2.92 | 299 | 17.44 | 16.36~18.5 | 467 | 17.8 | 16.7~19 |
| -1 | Survivors | 759 | 0.85 | 0.79~0.91 | 571 | 12.84 | 12.68~13.01 | 1200 | 10.6 | 10.4~10.9 |
| -1 | Non-survivors | 301 | 2.86 | 2.46~3.26 | 311 | 17.65 | 16.99~18.31 | 478 | 17.8 | 16.8~18.9 |
| 0 | Survivors | 1107 | 0.73 | 0.68~0.78 | 649 | 12.82 | 12.68~12.95 | 1658 | 9.7 | 9.5~9.9 |
| 0 | Non-survivors | 281 | 3.38 | 2.88~3.85 | 280 | 20.43 | 18.54~22.38 | 470 | 16.0 | 14.9~17.1 |

N, number of measurements; CI, confidence interval; TB, total bilirubin; PT, prothrombin time; WBC, white blood cell
