# Supplementary material for: Trajectories of longitudinal biomarkers for mortality in severely burned patients
Source: Sci Rep. 2020 Oct 1;10:16193. doi: 10.1038/s41598-020-73286-8 (PMC7530734; doi:10.1038/s41598-020-73286-8)
Supplement: Supplementary file 2 — Supplementary Table S2. [file 41598_2020_73286_MOESM2_ESM.docx]

**Trajectories of longitudinal biomarkers for mortality in severely burned patients**

Running title: Trajectories of longitudinal biomarkers in burns

Jaechul Yoon^1,2^, Dohern Kym^1+^, Jae Hee Won^1^, Jun Hur^1^*, Haejun Yim^1^, Yong Suk Cho^1^, Wook Chun^1^

^1^Department of Surgery and Critical Care, Burn Center, Hangang Sacred Heart Hospital, College of Medicine, Hallym University Medical, Seoul, Republic of Korea

^2^ Graduate school of Medicine, Kanwon National University, Chuncheon, Republic of Korea

^+^ This author contributed equally to this work as a first author. ORCID: 0000-0001-9178-0599

*Corresponding authors

Department of Surgery and Critical Care, Burn Center, Hangang Sacred Heart Hospital, College of Medicine, Hallym University **12, Beodeunaru-ro 7-gil**, Youngdeungpo-gu, Seoul, Korea, 07247

Tel. 82-2-2639-5446, Fax. 82-2-2678-4386, E-mail: [hammerj@hallym.or.kr](mailto:hammerj@hallym.or.kr)

1. Supplementary Table S2.The number of measurements, mean, CI in Forward

A) Platelet, Lactate, Creatinine in Forward

| Days | Group | Platetlet | | Lactate | | Creatinine | |
| --- | --- | --- | --- | --- | --- | --- | --- |
|  |  | Fitted value | 95% CI | Fitted value | 95% CI | Fitted value | 95% CI |
| 0 | Survivors | 200.9 | 197.8~203.9 | 2.20 | 2.16~2.25 | 0.77 | 0.76~0.79 |
| 0 | Non-survivors | 141.5 | 136.6~146.6 | 4.00 | 3.82~4.18 | 1.17 | 1.09~1.25 |
| 1 | Survivors | 211.9 | 209.3~214.9 | 2.13 | 2.08~2.17 | 0.76 | 0.75~0.78 |
| 1 | Non-survivors | 138.7 | 133.7~143.8 | 3.90 | 3.7~4.1 | 1.21 | 1.14~1.29 |
| 2 | Survivors | 222.5 | 219.5~225.6 | 2.08 | 2.03~2.13 | 0.76 | 0.74~0.78 |
| 2 | Non-survivors | 136.1 | 131.3~141.3 | 3.66 | 3.48~3.83 | 1.25 | 1.17~1.33 |
| 3 | Survivors | 233.5 | 230.2~236.7 | 2.04 | 1.99~2.08 | 0.75 | 0.73~0.78 |
| 3 | Non-survivors | 132.4 | 127.2~137.8 | 3.36 | 3.22~3.5 | 1.25 | 1.16~1.34 |
| 4 | Survivors | 245.4 | 241.7~248.8 | 1.99 | 1.95~2.04 | 0.74 | 0.72~0.76 |
| 4 | Non-survivors | 130.9 | 125.5~136.5 | 3.20 | 3.07~3.33 | 1.23 | 1.14~1.33 |
| 5 | Survivors | 257.7 | 253.7~261.5 | 2.00 | 1.95~2.05 | 0.74 | 0.71~0.76 |
| 5 | Non-survivors | 130.1 | 123.7~136.3 | 3.00 | 2.88~3.13 | 1.23 | 1.15~1.32 |
| 6 | Survivors | 271.1 | 266.7~275.3 | 1.92 | 1.88~1.97 | 0.73 | 0.71~0.76 |
| 6 | Non-survivors | 131.0 | 124.2~137.5 | 2.93 | 2.81~3.05 | 1.21 | 1.12~1.29 |
| 7 | Survivors | 283.8 | 279~288.6 | 1.91 | 1.86~1.96 | 0.73 | 0.7~0.76 |
| 7 | Non-survivors | 130.1 | 123~137.3 | 2.83 | 2.72~2.94 | 1.19 | 1.11~1.28 |
| 8 | Survivors | 295.7 | 290.3~300.8 | 1.90 | 1.85~1.94 | 0.73 | 0.7~0.75 |
| 8 | Non-survivors | 130.0 | 122.4~137.9 | 2.78 | 2.66~2.89 | 1.17 | 1.1~1.25 |
| 9 | Survivors | 308.6 | 302.7~314.2 | 1.87 | 1.82~1.92 | 0.73 | 0.7~0.76 |
| 9 | Non-survivors | 131.4 | 123~139.8 | 2.70 | 2.59~2.81 | 1.17 | 1.09~1.25 |
| 10 | Survivors | 319.2 | 313~325.9 | 1.85 | 1.8~1.9 | 0.72 | 0.69~0.75 |
| 10 | Non-survivors | 136.1 | 127.1~145 | 2.58 | 2.47~2.69 | 1.17 | 1.08~1.25 |
| 11 | Survivors | 330.8 | 323.6~337.5 | 1.84 | 1.8~1.89 | 0.72 | 0.7~0.76 |
| 11 | Non-survivors | 140.6 | 130.4~150.1 | 2.57 | 2.46~2.68 | 1.14 | 1.05~1.23 |
| 12 | Survivors | 338.1 | 330.4~345.8 | 1.82 | 1.77~1.86 | 0.72 | 0.68~0.75 |
| 12 | Non-survivors | 146.7 | 136~157.1 | 2.51 | 2.39~2.62 | 1.11 | 1.02~1.21 |
| 13 | Survivors | 346.0 | 337.2~354 | 1.78 | 1.73~1.82 | 0.72 | 0.68~0.75 |
| 13 | Non-survivors | 155.2 | 143.7~166.4 | 2.52 | 2.41~2.64 | 1.05 | 0.97~1.13 |
| 14 | Survivors | 353.1 | 343.8~362 | 1.79 | 1.74~1.84 | 0.71 | 0.68~0.75 |
| 14 | Non-survivors | 156.9 | 144.9~169.3 | 2.53 | 2.4~2.66 | 1.06 | 0.97~1.14 |
| 15 | Survivors | 356.0 | 346.8~365.2 | 1.79 | 1.75~1.84 | 0.72 | 0.68~0.76 |
| 15 | Non-survivors | 159.0 | 146.9~171.1 | 2.53 | 2.39~2.67 | 1.06 | 0.97~1.15 |
| 16 | Survivors | 357.8 | 347.7~367.5 | 1.78 | 1.73~1.84 | 0.71 | 0.67~0.75 |
| 16 | Non-survivors | 161.7 | 148.5~174.6 | 2.60 | 2.44~2.76 | 1.06 | 0.97~1.16 |
| 17 | Survivors | 364.4 | 353.8~374.5 | 1.75 | 1.69~1.8 | 0.71 | 0.67~0.75 |
| 17 | Non-survivors | 161.4 | 148.2~174.8 | 2.59 | 2.41~2.76 | 1.06 | 0.97~1.15 |
| 18 | Survivors | 367.1 | 356.4~377.9 | 1.76 | 1.71~1.82 | 0.71 | 0.67~0.75 |
| 18 | Non-survivors | 168.8 | 153.7~183.5 | 2.62 | 2.44~2.79 | 1.01 | 0.92~1.09 |
| 19 | Survivors | 366.8 | 355.7~378.1 | 1.71 | 1.66~1.76 | 0.72 | 0.67~0.76 |
| 19 | Non-survivors | 167.9 | 152.8~183.2 | 2.63 | 2.44~2.83 | 1.01 | 0.92~1.11 |
| 20 | Survivors | 370.4 | 359.1~381.7 | 1.73 | 1.67~1.78 | 0.71 | 0.67~0.76 |
| 20 | Non-survivors | 168.5 | 152.6~183.3 | 2.63 | 2.45~2.83 | 1.03 | 0.93~1.14 |
| 21 | Survivors | 371.7 | 359.8~383.5 | 1.74 | 1.69~1.8 | 0.73 | 0.68~0.78 |
| 21 | Non-survivors | 170.6 | 154.5~187.3 | 2.70 | 2.5~2.92 | 0.99 | 0.89~1.08 |
| 22 | Survivors | 370.4 | 358.5~382.2 | 1.72 | 1.67~1.78 | 0.73 | 0.68~0.78 |
| 22 | Non-survivors | 166.4 | 150.3~182.8 | 2.72 | 2.48~2.96 | 0.99 | 0.88~1.09 |
| 23 | Survivors | 365.4 | 353.7~377 | 1.67 | 1.61~1.73 | 0.74 | 0.69~0.8 |
| 23 | Non-survivors | 169.5 | 152.4~186.2 | 2.73 | 2.47~2.99 | 1.00 | 0.89~1.1 |
| 24 | Survivors | 369.3 | 356.2~381.4 | 1.65 | 1.6~1.71 | 0.74 | 0.69~0.8 |
| 24 | Non-survivors | 162.4 | 144.9~179.8 | 2.75 | 2.49~3 | 1.00 | 0.89~1.1 |
| 25 | Survivors | 364.9 | 352.7~377.8 | 1.70 | 1.63~1.76 | 0.75 | 0.69~0.81 |
| 25 | Non-survivors | 165.5 | 145.6~185 | 2.69 | 2.44~2.94 | 1.00 | 0.9~1.1 |
| 26 | Survivors | 366.2 | 353.3~378.8 | 1.65 | 1.59~1.72 | 0.73 | 0.69~0.77 |
| 26 | Non-survivors | 166.2 | 146.6~185.6 | 2.69 | 2.42~2.98 | 1.00 | 0.89~1.1 |
| 27 | Survivors | 370.3 | 356.8~384 | 1.65 | 1.58~1.72 | 0.73 | 0.69~0.78 |
| 27 | Non-survivors | 172.4 | 151~193.4 | 2.81 | 2.52~3.1 | 0.95 | 0.85~1.06 |
| 28 | Survivors | 375.0 | 361.2~389 | 1.69 | 1.62~1.75 | 0.73 | 0.69~0.78 |
| 28 | Non-survivors | 166.8 | 146.5~186.9 | 2.73 | 2.41~3.05 | 0.94 | 0.84~1.05 |
| 29 | Survivors | 371.6 | 357.7~385.6 | 1.68 | 1.61~1.75 | 0.75 | 0.7~0.8 |
| 29 | Non-survivors | 169.8 | 147.9~193.6 | 2.81 | 2.48~3.13 | 0.95 | 0.84~1.06 |
| 30 | Survivors | 365.1 | 350.4~379.4 | 1.68 | 1.62~1.75 | 0.74 | 0.69~0.78 |
| 30 | Non-survivors | 164.5 | 141.4~188.2 | 2.85 | 2.52~3.18 | 0.95 | 0.83~1.06 |
| 31 | Survivors | 371.7 | 354.8~387.3 | 1.66 | 1.58~1.73 | 0.78 | 0.73~0.84 |
| 31 | Non-survivors | 158.0 | 134.3~180.3 | 3.01 | 2.64~3.38 | 0.92 | 0.82~1.03 |
| 32 | Survivors | 368.2 | 353.3~383.8 | 1.66 | 1.58~1.74 | 0.78 | 0.72~0.83 |
| 32 | Non-survivors | 166.1 | 140.1~191.6 | 2.93 | 2.56~3.32 | 0.96 | 0.84~1.08 |
| 33 | Survivors | 365.3 | 350.1~380 | 1.58 | 1.5~1.65 | 0.78 | 0.72~0.83 |
| 33 | Non-survivors | 167.7 | 139.7~195.2 | 2.91 | 2.49~3.33 | 0.92 | 0.81~1.04 |
| 34 | Survivors | 364.5 | 347.6~380.6 | 1.64 | 1.57~1.72 | 0.80 | 0.74~0.85 |
| 34 | Non-survivors | 163.0 | 137.2~188.9 | 2.98 | 2.57~3.42 | 0.98 | 0.84~1.1 |
| 35 | Survivors | 363.5 | 347.2~380.1 | 1.59 | 1.52~1.67 | 0.78 | 0.72~0.84 |
| 35 | Non-survivors | 170.5 | 141.4~200.6 | 3.06 | 2.53~3.59 | 0.97 | 0.83~1.1 |
| 36 | Survivors | 360.3 | 343~378 | 1.66 | 1.57~1.74 | 0.80 | 0.74~0.86 |
| 36 | Non-survivors | 169.5 | 139.1~198.9 | 2.90 | 2.43~3.34 | 0.94 | 0.82~1.08 |
| 37 | Survivors | 355.2 | 338.9~372.9 | 1.61 | 1.53~1.69 | 0.79 | 0.73~0.85 |
| 37 | Non-survivors | 178.5 | 146.2~210.3 | 2.91 | 2.44~3.4 | 0.94 | 0.81~1.08 |
| 38 | Survivors | 347.0 | 328.2~365.7 | 1.65 | 1.55~1.75 | 0.80 | 0.74~0.86 |
| 38 | Non-survivors | 165.7 | 135.7~194.9 | 2.94 | 2.41~3.46 | 0.91 | 0.76~1.05 |
| 39 | Survivors | 345.5 | 327~362.8 | 1.65 | 1.56~1.74 | 0.78 | 0.72~0.83 |
| 39 | Non-survivors | 176.5 | 138.4~213.5 | 3.01 | 2.45~3.58 | 0.88 | 0.72~1.03 |
| 40 | Survivors | 351.5 | 332.2~369.5 | 1.60 | 1.51~1.7 | 0.78 | 0.72~0.84 |
| 40 | Non-survivors | 181.4 | 145.2~219.2 | 2.97 | 2.3~3.61 | 0.84 | 0.7~0.97 |
| 41 | Survivors | 347.1 | 327.5~367.2 | 1.63 | 1.53~1.73 | 0.79 | 0.73~0.86 |
| 41 | Non-survivors | 184.3 | 144.1~225.3 | 2.96 | 2.33~3.6 | 0.87 | 0.72~1.01 |
| 42 | Survivors | 341.1 | 320.2~361.3 | 1.67 | 1.57~1.77 | 0.80 | 0.73~0.86 |
| 42 | Non-survivors | 181.0 | 138.9~224.2 | 3.03 | 2.39~3.66 | 0.85 | 0.7~1 |
| 43 | Survivors | 341.1 | 320.3~361.6 | 1.59 | 1.49~1.69 | 0.82 | 0.74~0.89 |
| 43 | Non-survivors | 162.6 | 126.6~198.4 | 3.01 | 2.36~3.68 | 0.88 | 0.71~1.04 |
| 44 | Survivors | 340.8 | 319~362.2 | 1.60 | 1.5~1.69 | 0.76 | 0.7~0.83 |
| 44 | Non-survivors | 189.0 | 146.7~228.6 | 2.99 | 2.32~3.64 | 0.86 | 0.7~1.01 |
| 45 | Survivors | 343.7 | 318.7~366.7 | 1.66 | 1.55~1.77 | 0.79 | 0.72~0.86 |
| 45 | Non-survivors | 177.8 | 137.2~215.8 | 2.93 | 2.2~3.73 | 0.83 | 0.67~0.99 |
| 46 | Survivors | 348.1 | 324.9~371.6 | 1.67 | 1.56~1.79 | 0.80 | 0.72~0.87 |
| 46 | Non-survivors | 195.4 | 146.6~245.5 | 2.70 | 1.95~3.44 | 0.88 | 0.69~1.08 |
| 47 | Survivors | 339.6 | 313.9~363.4 | 1.63 | 1.5~1.76 | 0.83 | 0.75~0.92 |
| 47 | Non-survivors | 169.4 | 124.4~214.4 | 2.98 | 2.12~3.88 | 0.85 | 0.65~1.04 |
| 48 | Survivors | 338.2 | 313.8~362.4 | 1.66 | 1.54~1.79 | 0.80 | 0.72~0.88 |
| 48 | Non-survivors | 180.2 | 132.6~228.4 | 3.22 | 2.15~4.25 | 0.91 | 0.68~1.13 |
| 49 | Survivors | 338.8 | 313.3~363.7 | 1.61 | 1.46~1.76 | 0.81 | 0.73~0.9 |
| 49 | Non-survivors | 210.2 | 150.7~269.3 | 3.12 | 2.07~4.25 | 0.84 | 0.66~1.04 |
| 50 | Survivors | 339.5 | 314~364.1 | 1.65 | 1.5~1.8 | 0.82 | 0.74~0.9 |
| 50 | Non-survivors | 188.4 | 139.6~239.4 | 2.72 | 1.84~3.63 | 0.82 | 0.57~1.06 |
| 51 | Survivors | 334.2 | 306.5~362.3 | 1.56 | 1.43~1.68 | 0.85 | 0.75~0.94 |
| 51 | Non-survivors | 189.9 | 139.7~241.9 | 2.25 | 1.78~2.72 | 0.88 | 0.63~1.12 |
| 52 | Survivors | 337.4 | 308.5~365.9 | 1.58 | 1.44~1.72 | 0.80 | 0.7~0.89 |
| 52 | Non-survivors | 207.3 | 147.4~268.3 | 2.61 | 1.95~3.25 | 0.70 | 0.51~0.9 |
| 53 | Survivors | 329.0 | 297.8~358.9 | 1.68 | 1.52~1.84 | 0.78 | 0.68~0.87 |
| 53 | Non-survivors | 196.4 | 135~255.1 | 2.05 | 1.58~2.49 | 0.84 | 0.59~1.08 |
| 54 | Survivors | 340.0 | 309.1~371.2 | 1.67 | 1.49~1.84 | 0.80 | 0.69~0.91 |
| 54 | Non-survivors | 194.0 | 137.8~248.5 | 2.67 | 1.94~3.4 | 0.76 | 0.5~1 |
| 55 | Survivors | 329.4 | 299.8~360.5 | 1.55 | 1.38~1.74 | 0.80 | 0.68~0.91 |
| 55 | Non-survivors | 209.7 | 144.3~279.3 | 2.68 | 1.93~3.48 | 0.78 | 0.54~1.01 |
| 56 | Survivors | 347.9 | 316.1~380.6 | 1.61 | 1.42~1.79 | 0.81 | 0.7~0.93 |
| 56 | Non-survivors | 232.2 | 159.3~303.7 | 2.19 | 1.75~2.63 | 0.78 | 0.52~1.02 |

N, number of measurements; CI, confidence interval

B) TB, PT, WBC in Forward

| Days | Group | TB | | PT | | WBC | |
| --- | --- | --- | --- | --- | --- | --- | --- |
|  |  | Fitted value | 95% CI | Fitted value | 95% CI | Fitted value | 95% CI |
| 0 | Survivors | 1.01 | 0.99~1.03 | 12.85 | 12.79~12.92 | 13.2 | 13.1~13.4 |
| 0 | Non-survivors | 0.98 | 0.92~1.05 | 15.07 | 14.78~15.36 | 17.0 | 16.5~17.5 |
| 1 | Survivors | 1.00 | 0.98~1.02 | 12.87 | 12.79~12.95 | 13.1 | 13~13.3 |
| 1 | Non-survivors | 1.09 | 1.03~1.16 | 15.46 | 15.04~15.86 | 16.9 | 16.4~17.4 |
| 2 | Survivors | 1.01 | 0.98~1.03 | 12.97 | 12.88~13.07 | 13.0 | 12.9~13.2 |
| 2 | Non-survivors | 1.16 | 1.08~1.24 | 15.43 | 15.03~15.83 | 16.7 | 16.2~17.1 |
| 3 | Survivors | 0.99 | 0.96~1.02 | 13.01 | 12.91~13.11 | 13.0 | 12.8~13.2 |
| 3 | Non-survivors | 1.24 | 1.15~1.34 | 15.06 | 14.79~15.34 | 16.3 | 15.8~16.7 |
| 4 | Survivors | 0.98 | 0.95~1.01 | 13.05 | 12.95~13.15 | 12.9 | 12.7~13.1 |
| 4 | Non-survivors | 1.29 | 1.2~1.39 | 15.06 | 14.82~15.31 | 16.1 | 15.7~16.5 |
| 5 | Survivors | 0.98 | 0.95~1.02 | 13.08 | 12.98~13.18 | 12.9 | 12.7~13.1 |
| 5 | Non-survivors | 1.40 | 1.28~1.52 | 15.16 | 14.88~15.43 | 16.0 | 15.6~16.5 |
| 6 | Survivors | 0.97 | 0.93~1.01 | 13.06 | 12.96~13.16 | 12.9 | 12.7~13.1 |
| 6 | Non-survivors | 1.52 | 1.36~1.68 | 15.13 | 14.85~15.43 | 15.9 | 15.4~16.3 |
| 7 | Survivors | 0.97 | 0.94~1.01 | 13.16 | 13.05~13.27 | 12.9 | 12.7~13.1 |
| 7 | Non-survivors | 1.56 | 1.39~1.73 | 15.23 | 14.92~15.54 | 15.8 | 15.3~16.2 |
| 8 | Survivors | 0.96 | 0.92~1 | 13.12 | 13.01~13.22 | 12.8 | 12.6~13 |
| 8 | Non-survivors | 1.79 | 1.57~2.02 | 15.46 | 15.13~15.79 | 15.6 | 15.2~16.1 |
| 9 | Survivors | 0.93 | 0.89~0.97 | 13.15 | 13.04~13.27 | 12.8 | 12.6~13 |
| 9 | Non-survivors | 1.82 | 1.58~2.06 | 15.48 | 15.14~15.82 | 15.5 | 15~16 |
| 10 | Survivors | 0.94 | 0.9~0.98 | 13.21 | 13.1~13.32 | 12.8 | 12.6~13 |
| 10 | Non-survivors | 1.88 | 1.6~2.16 | 15.62 | 15.28~15.96 | 15.4 | 14.8~15.9 |
| 11 | Survivors | 0.91 | 0.87~0.95 | 13.21 | 13.1~13.32 | 12.7 | 12.5~12.9 |
| 11 | Non-survivors | 2.03 | 1.74~2.33 | 15.63 | 15.3~15.95 | 15.4 | 14.9~15.9 |
| 12 | Survivors | 0.92 | 0.87~0.96 | 13.21 | 13.1~13.32 | 12.6 | 12.4~12.8 |
| 12 | Non-survivors | 1.91 | 1.65~2.17 | 15.37 | 15.04~15.74 | 15.3 | 14.7~15.8 |
| 13 | Survivors | 0.90 | 0.85~0.94 | 13.22 | 13.11~13.34 | 12.6 | 12.3~12.8 |
| 13 | Non-survivors | 1.86 | 1.6~2.13 | 15.41 | 15.11~15.71 | 15.2 | 14.6~15.7 |
| 14 | Survivors | 0.90 | 0.85~0.95 | 13.41 | 13.29~13.53 | 12.5 | 12.3~12.7 |
| 14 | Non-survivors | 1.96 | 1.69~2.23 | 15.67 | 15.36~15.99 | 15.3 | 14.7~15.9 |
| 15 | Survivors | 0.88 | 0.83~0.93 | 13.47 | 13.34~13.59 | 12.3 | 12.1~12.5 |
| 15 | Non-survivors | 2.14 | 1.86~2.45 | 15.79 | 15.37~16.19 | 15.3 | 14.7~15.8 |
| 16 | Survivors | 0.90 | 0.84~0.95 | 13.54 | 13.41~13.66 | 12.3 | 12.1~12.5 |
| 16 | Non-survivors | 2.05 | 1.72~2.39 | 15.84 | 15.39~16.3 | 15.2 | 14.6~15.8 |
| 17 | Survivors | 0.91 | 0.85~0.97 | 13.51 | 13.37~13.63 | 12.2 | 11.9~12.4 |
| 17 | Non-survivors | 2.06 | 1.76~2.35 | 15.96 | 15.55~16.39 | 15.0 | 14.5~15.7 |
| 18 | Survivors | 0.88 | 0.83~0.92 | 13.44 | 13.31~13.57 | 12.1 | 11.8~12.3 |
| 18 | Non-survivors | 2.21 | 1.82~2.6 | 15.90 | 15.53~16.26 | 15.1 | 14.4~15.9 |
| 19 | Survivors | 0.87 | 0.81~0.92 | 13.46 | 13.32~13.59 | 12.0 | 11.7~12.2 |
| 19 | Non-survivors | 2.15 | 1.74~2.56 | 16.20 | 15.6~16.78 | 15.2 | 14.5~16 |
| 20 | Survivors | 0.84 | 0.79~0.89 | 13.57 | 13.41~13.71 | 11.8 | 11.6~12.1 |
| 20 | Non-survivors | 2.54 | 2.06~3.02 | 16.16 | 15.7~16.59 | 15.3 | 14.4~16.1 |
| 21 | Survivors | 0.89 | 0.82~0.95 | 13.61 | 13.45~13.77 | 11.8 | 11.6~12.1 |
| 21 | Non-survivors | 2.42 | 1.96~2.9 | 16.48 | 16.04~16.94 | 15.2 | 14.2~16.1 |
| 22 | Survivors | 0.83 | 0.78~0.88 | 13.56 | 13.39~13.73 | 11.6 | 11.4~11.9 |
| 22 | Non-survivors | 2.55 | 2.03~3.05 | 16.85 | 16.2~17.51 | 15.5 | 14.4~16.5 |
| 23 | Survivors | 0.88 | 0.81~0.94 | 13.50 | 13.31~13.68 | 11.6 | 11.3~11.9 |
| 23 | Non-survivors | 2.89 | 2.18~3.57 | 16.46 | 15.95~16.97 | 15.6 | 14.5~16.7 |
| 24 | Survivors | 0.91 | 0.83~0.99 | 13.49 | 13.31~13.67 | 11.6 | 11.4~11.9 |
| 24 | Non-survivors | 2.67 | 2.08~3.27 | 16.96 | 16.38~17.52 | 15.6 | 14.4~16.8 |
| 25 | Survivors | 0.88 | 0.8~0.96 | 13.56 | 13.39~13.76 | 11.5 | 11.2~11.8 |
| 25 | Non-survivors | 2.91 | 2.13~3.67 | 16.58 | 15.86~17.23 | 15.6 | 14.3~16.9 |
| 26 | Survivors | 0.88 | 0.79~0.97 | 13.51 | 13.31~13.71 | 11.5 | 11.2~11.7 |
| 26 | Non-survivors | 2.89 | 2.19~3.64 | 17.07 | 15.99~18.13 | 15.8 | 14.3~17.1 |
| 27 | Survivors | 0.87 | 0.78~0.96 | 13.60 | 13.4~13.8 | 11.4 | 11.2~11.7 |
| 27 | Non-survivors | 2.60 | 1.96~3.21 | 16.64 | 15.88~17.36 | 15.2 | 13.8~16.5 |
| 28 | Survivors | 0.90 | 0.8~1 | 13.57 | 13.37~13.79 | 11.3 | 11~11.6 |
| 28 | Non-survivors | 2.82 | 2.04~3.58 | 17.10 | 16.24~17.95 | 15.4 | 14.1~16.9 |
| 29 | Survivors | 0.88 | 0.77~0.98 | 13.78 | 13.54~14.02 | 11.3 | 11~11.6 |
| 29 | Non-survivors | 3.08 | 2.17~4 | 17.25 | 16.41~18.09 | 15.6 | 14~17.1 |
| 30 | Survivors | 0.89 | 0.79~0.99 | 13.72 | 13.5~13.94 | 11.2 | 10.9~11.5 |
| 30 | Non-survivors | 3.04 | 2.06~3.95 | 17.34 | 16.36~18.29 | 15.9 | 14.2~17.6 |
| 31 | Survivors | 0.87 | 0.76~0.98 | 13.75 | 13.51~13.97 | 11.0 | 10.8~11.4 |
| 31 | Non-survivors | 2.91 | 2.08~3.78 | 17.29 | 16.27~18.3 | 15.8 | 14.1~17.5 |
| 32 | Survivors | 0.90 | 0.78~1.02 | 13.76 | 13.51~14 | 11.1 | 10.8~11.4 |
| 32 | Non-survivors | 2.90 | 1.92~3.9 | 17.31 | 16.13~18.41 | 16.0 | 14.2~17.9 |
| 33 | Survivors | 0.93 | 0.78~1.06 | 13.74 | 13.45~14.03 | 10.8 | 10.5~11.1 |
| 33 | Non-survivors | 3.23 | 2.13~4.33 | 17.79 | 16.58~18.98 | 15.3 | 13.4~17.1 |
| 34 | Survivors | 0.87 | 0.73~1 | 13.77 | 13.47~14.06 | 10.9 | 10.6~11.2 |
| 34 | Non-survivors | 3.44 | 2.15~4.7 | 17.55 | 16.29~18.9 | 15.2 | 13~17.2 |
| 35 | Survivors | 0.89 | 0.74~1.05 | 13.81 | 13.51~14.1 | 10.7 | 10.4~11.1 |
| 35 | Non-survivors | 2.84 | 1.87~3.79 | 17.60 | 16.19~18.96 | 14.6 | 12.8~16.4 |
| 36 | Survivors | 0.92 | 0.75~1.09 | 13.73 | 13.38~14.1 | 10.7 | 10.4~11.1 |
| 36 | Non-survivors | 3.00 | 1.92~4.02 | 17.56 | 16.16~18.99 | 14.6 | 12.7~16.4 |
| 37 | Survivors | 0.91 | 0.74~1.08 | 13.67 | 13.31~14.03 | 10.5 | 10.1~10.9 |
| 37 | Non-survivors | 3.02 | 1.85~4.17 | 16.89 | 15.7~18.09 | 14.2 | 12.4~16 |
| 38 | Survivors | 0.90 | 0.71~1.09 | 13.78 | 13.46~14.11 | 10.4 | 10.1~10.8 |
| 38 | Non-survivors | 3.09 | 1.96~4.28 | 17.45 | 16.23~18.75 | 13.9 | 12~15.8 |
| 39 | Survivors | 0.98 | 0.78~1.18 | 13.90 | 13.5~14.31 | 10.4 | 10~10.7 |
| 39 | Non-survivors | 2.25 | 1.37~3.08 | 16.84 | 15.79~17.9 | 13.6 | 11.4~15.6 |
| 40 | Survivors | 1.03 | 0.82~1.27 | 13.85 | 13.44~14.27 | 10.3 | 10~10.7 |
| 40 | Non-survivors | 2.24 | 1.43~3.06 | 17.17 | 15.83~18.56 | 13.0 | 11.1~15 |
| 41 | Survivors | 1.01 | 0.79~1.24 | 13.85 | 13.49~14.23 | 10.2 | 9.8~10.6 |
| 41 | Non-survivors | 2.40 | 1.63~3.19 | 16.77 | 15.57~17.96 | 12.8 | 10.8~14.9 |
| 42 | Survivors | 1.04 | 0.76~1.3 | 13.78 | 13.41~14.11 | 10.2 | 9.8~10.6 |
| 42 | Non-survivors | 2.49 | 1.52~3.4 | 17.50 | 16.24~18.72 | 13.1 | 11~15.1 |
| 43 | Survivors | 0.84 | 0.69~1 | 13.79 | 13.44~14.14 | 10.1 | 9.7~10.6 |
| 43 | Non-survivors | 2.51 | 1.58~3.43 | 17.43 | 16.09~18.73 | 13.1 | 10.9~15.2 |
| 44 | Survivors | 0.84 | 0.68~0.99 | 13.67 | 13.32~14.02 | 10.0 | 9.5~10.4 |
| 44 | Non-survivors | 2.04 | 1.26~2.83 | 16.56 | 15.48~17.7 | 12.2 | 10.3~14 |
| 45 | Survivors | 1.00 | 0.7~1.3 | 13.55 | 13.25~13.88 | 10.1 | 9.6~10.6 |
| 45 | Non-survivors | 2.71 | 1.82~3.55 | 17.07 | 15.78~18.38 | 12.3 | 10.3~14.3 |
| 46 | Survivors | 1.06 | 0.75~1.37 | 13.79 | 13.42~14.15 | 10.1 | 9.6~10.6 |
| 46 | Non-survivors | 2.29 | 1.34~3.22 | 16.43 | 15.08~17.82 | 12.3 | 10.4~14.4 |
| 47 | Survivors | 1.04 | 0.66~1.4 | 13.92 | 13.51~14.33 | 10.0 | 9.4~10.5 |
| 47 | Non-survivors | 2.16 | 1.1~3.19 | 17.04 | 15.46~18.62 | 11.4 | 9.6~13.2 |
| 48 | Survivors | 1.02 | 0.72~1.33 | 13.78 | 13.39~14.17 | 9.7 | 9.2~10.2 |
| 48 | Non-survivors | 2.52 | 1.16~3.86 | 17.85 | 16.08~19.65 | 11.4 | 9.3~13.3 |
| 49 | Survivors | 1.07 | 0.72~1.46 | 14.04 | 13.4~14.64 | 9.6 | 9~10.1 |
| 49 | Non-survivors | 2.08 | 1.1~3.05 | 17.78 | 15.92~19.68 | 11.2 | 9.4~13.1 |
| 50 | Survivors | 1.16 | 0.73~1.57 | 14.09 | 13.34~14.79 | 9.5 | 9~10.1 |
| 50 | Non-survivors | 2.73 | 1.19~4.23 | 16.93 | 14.86~19.13 | 11.2 | 9.1~13.3 |
| 51 | Survivors | 1.15 | 0.7~1.6 | 13.97 | 13.45~14.5 | 9.5 | 8.8~10.1 |
| 51 | Non-survivors | 2.13 | 0.71~3.45 | 16.13 | 14.41~17.93 | 11.0 | 8.7~13.4 |
| 52 | Survivors | 1.13 | 0.67~1.6 | 14.24 | 13.34~15.14 | 9.6 | 9~10.2 |
| 52 | Non-survivors | 2.29 | 1.02~3.6 | 17.84 | 15.55~20.05 | 10.9 | 8.6~13 |
| 53 | Survivors | 1.26 | 0.77~1.79 | 14.18 | 13.59~14.74 | 9.7 | 9~10.4 |
| 53 | Non-survivors | 2.22 | 0.99~3.46 | 16.73 | 14.45~18.93 | 11.0 | 8.5~13.5 |
| 54 | Survivors | 1.04 | 0.69~1.39 | 14.01 | 13.48~14.57 | 9.6 | 8.9~10.3 |
| 54 | Non-survivors | 2.32 | 0.72~3.93 | 16.83 | 14.22~19.28 | 10.9 | 8.3~13.3 |
| 55 | Survivors | 0.91 | 0.61~1.17 | 13.96 | 13.35~14.54 | 9.5 | 8.8~10.2 |
| 55 | Non-survivors | 2.52 | 0.93~4.06 | 17.02 | 14.77~19.29 | 10.9 | 8.3~13.5 |
| 56 | Survivors | 1.43 | 0.76~2.12 | 13.75 | 13.25~14.24 | 9.3 | 8.6~10 |
| 56 | Non-survivors | 2.17 | 0.81~3.53 | 17.37 | 15.11~19.69 | 10.6 | 8.2~13.2 |

N, number of measurements; CI, confidence intreval

2. Tables for the number of measurements, mean, CI in Backward

A) Platelet, Lactate, Creatinine in Backward

| Days | Group | Plactelet | | Lactate | | Creatinine | |
| --- | --- | --- | --- | --- | --- | --- | --- |
|  |  | Fitted value | 95% CI | Fitted value | 95% CI | Fitted value | 95% CI |
| -56 | Survivors | 255.7 | 236.8~274.1 | 2.28 | 2.12~2.44 | 0.81 | 0.71~0.91 |
| -56 | Non-survivors | 252.8 | 213.3~293.5 | 2.08 | 1.82~2.33 | 0.85 | 0.66~1.04 |
| -55 | Survivors | 256.6 | 239.9~273 | 2.19 | 2.05~2.32 | 0.84 | 0.74~0.94 |
| -55 | Non-survivors | 245.2 | 204.9~284.4 | 2.18 | 1.84~2.53 | 0.85 | 0.67~1.02 |
| -54 | Survivors | 256.3 | 239.6~273.6 | 2.24 | 2.09~2.39 | 0.85 | 0.75~0.95 |
| -54 | Non-survivors | 244.6 | 205~283.6 | 2.21 | 1.9~2.54 | 0.79 | 0.6~0.98 |
| -53 | Survivors | 257.6 | 241.7~273.6 | 2.25 | 2.09~2.39 | 0.84 | 0.74~0.92 |
| -53 | Non-survivors | 241.8 | 197.5~285.6 | 2.16 | 1.82~2.48 | 0.79 | 0.63~0.95 |
| -52 | Survivors | 264.2 | 249.3~279.8 | 2.13 | 2~2.27 | 0.83 | 0.74~0.92 |
| -52 | Non-survivors | 237.8 | 196.9~276.2 | 2.15 | 1.82~2.49 | 0.78 | 0.61~0.95 |
| -51 | Survivors | 266.3 | 251.1~282.5 | 2.15 | 2.01~2.29 | 0.82 | 0.74~0.9 |
| -51 | Non-survivors | 232.0 | 191.9~273.2 | 2.10 | 1.78~2.41 | 0.78 | 0.6~0.97 |
| -50 | Survivors | 268.9 | 254.6~283.8 | 2.12 | 1.98~2.26 | 0.82 | 0.74~0.9 |
| -50 | Non-survivors | 229.4 | 193.5~265.4 | 2.16 | 1.91~2.41 | 0.87 | 0.69~1.04 |
| -49 | Survivors | 272.6 | 258.3~287 | 2.06 | 1.93~2.2 | 0.81 | 0.74~0.88 |
| -49 | Non-survivors | 229.5 | 197.4~263.6 | 2.00 | 1.69~2.29 | 0.84 | 0.65~1.01 |
| -48 | Survivors | 273.3 | 258.8~287.1 | 2.01 | 1.9~2.12 | 0.81 | 0.73~0.88 |
| -48 | Non-survivors | 228.8 | 195.2~262.2 | 2.03 | 1.72~2.35 | 0.83 | 0.64~1 |
| -47 | Survivors | 276.2 | 261.3~290.5 | 2.01 | 1.9~2.11 | 0.82 | 0.75~0.89 |
| -47 | Non-survivors | 226.6 | 195.3~259.7 | 2.08 | 1.76~2.41 | 0.90 | 0.68~1.13 |
| -46 | Survivors | 274.9 | 261.7~288.6 | 2.05 | 1.94~2.16 | 0.80 | 0.73~0.86 |
| -46 | Non-survivors | 232.4 | 200.6~264 | 1.94 | 1.68~2.21 | 0.87 | 0.65~1.08 |
| -45 | Survivors | 276.8 | 263~290.5 | 2.06 | 1.96~2.18 | 0.81 | 0.74~0.87 |
| -45 | Non-survivors | 232.8 | 203.8~262 | 1.91 | 1.62~2.2 | 0.87 | 0.66~1.08 |
| -44 | Survivors | 275.6 | 263~288.1 | 2.00 | 1.91~2.1 | 0.80 | 0.74~0.86 |
| -44 | Non-survivors | 225.8 | 194.8~256.3 | 1.94 | 1.69~2.2 | 0.84 | 0.66~1.04 |
| -43 | Survivors | 279.0 | 266.4~291.5 | 2.01 | 1.91~2.11 | 0.80 | 0.74~0.86 |
| -43 | Non-survivors | 223.6 | 193.5~253.6 | 2.11 | 1.89~2.34 | 0.86 | 0.7~1.03 |
| -42 | Survivors | 279.6 | 267.1~292.8 | 2.05 | 1.95~2.14 | 0.79 | 0.74~0.84 |
| -42 | Non-survivors | 219.3 | 189.8~251.3 | 2.03 | 1.8~2.26 | 0.86 | 0.7~1.01 |
| -41 | Survivors | 281.3 | 268.8~293.8 | 2.03 | 1.93~2.13 | 0.80 | 0.74~0.85 |
| -41 | Non-survivors | 217.7 | 190.9~245.4 | 2.05 | 1.82~2.26 | 0.84 | 0.69~1 |
| -40 | Survivors | 283.8 | 271.2~296.6 | 2.06 | 1.96~2.15 | 0.79 | 0.74~0.84 |
| -40 | Non-survivors | 212.2 | 184.4~239.5 | 2.12 | 1.91~2.34 | 0.92 | 0.68~1.16 |
| -39 | Survivors | 282.0 | 269.4~294.1 | 2.05 | 1.95~2.15 | 0.77 | 0.72~0.82 |
| -39 | Non-survivors | 208.6 | 181.3~237.7 | 2.18 | 1.95~2.42 | 0.92 | 0.69~1.16 |
| -38 | Survivors | 283.9 | 272~295.8 | 2.04 | 1.95~2.13 | 0.77 | 0.73~0.81 |
| -38 | Non-survivors | 213.8 | 185.7~241.2 | 2.08 | 1.83~2.33 | 1.02 | 0.79~1.25 |
| -37 | Survivors | 283.8 | 271.9~294.9 | 2.01 | 1.93~2.1 | 0.77 | 0.73~0.81 |
| -37 | Non-survivors | 209.6 | 184.3~233.6 | 2.03 | 1.83~2.24 | 0.92 | 0.72~1.14 |
| -36 | Survivors | 285.6 | 274.5~297.3 | 2.03 | 1.95~2.1 | 0.76 | 0.72~0.8 |
| -36 | Non-survivors | 209.3 | 184.3~234.7 | 2.22 | 2.01~2.42 | 0.93 | 0.73~1.13 |
| -35 | Survivors | 285.3 | 275~296.2 | 2.05 | 1.96~2.12 | 0.76 | 0.72~0.8 |
| -35 | Non-survivors | 210.3 | 185.6~235.7 | 2.11 | 1.89~2.32 | 0.94 | 0.75~1.12 |
| -34 | Survivors | 284.4 | 274.2~295 | 2.02 | 1.95~2.09 | 0.77 | 0.72~0.8 |
| -34 | Non-survivors | 203.2 | 180.4~227.4 | 2.19 | 1.97~2.41 | 0.98 | 0.8~1.18 |
| -33 | Survivors | 287.2 | 276.5~297.6 | 1.99 | 1.92~2.06 | 0.75 | 0.72~0.79 |
| -33 | Non-survivors | 196.4 | 173.7~220.9 | 2.29 | 2.08~2.49 | 0.99 | 0.81~1.18 |
| -32 | Survivors | 287.3 | 277.2~297.6 | 1.98 | 1.92~2.05 | 0.77 | 0.73~0.8 |
| -32 | Non-survivors | 190.8 | 169.8~212.1 | 2.38 | 2.16~2.59 | 0.98 | 0.81~1.16 |
| -31 | Survivors | 289.5 | 279.5~299.6 | 1.98 | 1.91~2.05 | 0.76 | 0.73~0.8 |
| -31 | Non-survivors | 188.1 | 167.1~207.6 | 2.41 | 2.18~2.63 | 0.98 | 0.81~1.15 |
| -30 | Survivors | 291.1 | 281.6~300.9 | 1.95 | 1.89~2.02 | 0.76 | 0.73~0.8 |
| -30 | Non-survivors | 186.7 | 165.6~206.9 | 2.35 | 2.13~2.55 | 0.96 | 0.81~1.11 |
| -29 | Survivors | 290.5 | 280.3~300.1 | 1.97 | 1.9~2.03 | 0.77 | 0.74~0.81 |
| -29 | Non-survivors | 189.8 | 169.9~211.3 | 2.35 | 2.15~2.55 | 0.94 | 0.8~1.09 |
| -28 | Survivors | 294.3 | 284.7~303.9 | 1.95 | 1.89~2.01 | 0.76 | 0.72~0.79 |
| -28 | Non-survivors | 183.1 | 164.5~200.6 | 2.37 | 2.18~2.56 | 0.92 | 0.8~1.05 |
| -27 | Survivors | 292.1 | 282.9~301.7 | 1.96 | 1.89~2.02 | 0.76 | 0.73~0.79 |
| -27 | Non-survivors | 185.7 | 168~204.2 | 2.43 | 2.21~2.65 | 0.92 | 0.8~1.03 |
| -26 | Survivors | 290.9 | 281.7~300.1 | 1.94 | 1.88~2 | 0.77 | 0.73~0.8 |
| -26 | Non-survivors | 178.2 | 161.7~195.1 | 2.50 | 2.31~2.7 | 0.90 | 0.8~1.01 |
| -25 | Survivors | 294.9 | 285.9~304.6 | 1.94 | 1.88~1.99 | 0.77 | 0.74~0.81 |
| -25 | Non-survivors | 173.8 | 159.2~189.7 | 2.56 | 2.36~2.75 | 0.90 | 0.8~1 |
| -24 | Survivors | 294.0 | 285.1~303.4 | 1.95 | 1.9~2.01 | 0.78 | 0.74~0.81 |
| -24 | Non-survivors | 172.8 | 157.4~188.6 | 2.55 | 2.37~2.74 | 0.89 | 0.8~0.99 |
| -23 | Survivors | 294.1 | 284.8~304.1 | 1.93 | 1.88~1.99 | 0.78 | 0.74~0.81 |
| -23 | Non-survivors | 171.3 | 157.2~185.4 | 2.55 | 2.38~2.72 | 0.89 | 0.81~0.98 |
| -22 | Survivors | 292.9 | 283.4~302.1 | 1.91 | 1.86~1.97 | 0.78 | 0.74~0.816 |
| -22 | Non-survivors | 165.4 | 152.8~178.6 | 2.63 | 2.46~2.81 | 0.91 | 0.824~1 |
| -21 | Survivors | 298.8 | 290.1~307.4 | 1.89 | 1.84~1.94 | 0.76 | 0.72~0.79 |
| -21 | Non-survivors | 168.4 | 154.9~182.2 | 2.58 | 2.41~2.76 | 0.92 | 0.83~1 |
| -20 | Survivors | 296.2 | 287.3~305.2 | 1.90 | 1.85~1.95 | 0.77 | 0.73~0.81 |
| -20 | Non-survivors | 162.9 | 150.5~175.9 | 2.63 | 2.46~2.78 | 0.92 | 0.84~1 |
| -19 | Survivors | 297.6 | 288.6~306.6 | 1.90 | 1.85~1.96 | 0.76 | 0.73~0.8 |
| -19 | Non-survivors | 161.6 | 149.5~174.4 | 2.66 | 2.51~2.82 | 0.92 | 0.84~1 |
| -18 | Survivors | 301.0 | 292~310.2 | 1.87 | 1.82~1.92 | 0.76 | 0.73~0.8 |
| -18 | Non-survivors | 160.0 | 148.1~172.4 | 2.71 | 2.57~2.87 | 0.93 | 0.85~1.01 |
| -17 | Survivors | 305.4 | 296.9~313.7 | 1.86 | 1.81~1.91 | 0.75 | 0.72~0.78 |
| -17 | Non-survivors | 158.5 | 146.7~170.1 | 2.76 | 2.61~2.92 | 0.96 | 0.88~1.05 |
| -16 | Survivors | 309.1 | 300.6~318 | 1.84 | 1.8~1.89 | 0.76 | 0.72~0.79 |
| -16 | Non-survivors | 158.7 | 146.6~170.6 | 2.79 | 2.64~2.94 | 0.94 | 0.87~1.02 |
| -15 | Survivors | 309.6 | 300.8~318.2 | 1.82 | 1.77~1.87 | 0.76 | 0.72~0.79 |
| -15 | Non-survivors | 154.5 | 143.6~165.3 | 2.86 | 2.7~3.01 | 0.96 | 0.88~1.03 |
| -14 | Survivors | 317.3 | 308.8~325.8 | 1.80 | 1.76~1.85 | 0.73 | 0.7~0.76 |
| -14 | Non-survivors | 153.5 | 142.9~164.2 | 2.85 | 2.7~3.01 | 0.96 | 0.89~1.03 |
| -13 | Survivors | 319.1 | 310.3~328 | 1.78 | 1.73~1.83 | 0.75 | 0.72~0.78 |
| -13 | Non-survivors | 147.8 | 138.6~157.5 | 2.89 | 2.73~3.04 | 0.95 | 0.89~1.02 |
| -12 | Survivors | 323.2 | 314.9~331.9 | 1.78 | 1.73~1.83 | 0.74 | 0.71~0.78 |
| -12 | Non-survivors | 147.7 | 138.1~157.4 | 2.96 | 2.81~3.11 | 0.98 | 0.91~1.05 |
| -11 | Survivors | 327.1 | 319~335.3 | 1.78 | 1.74~1.83 | 0.74 | 0.7~0.77 |
| -11 | Non-survivors | 143.6 | 134.5~152.2 | 3.00 | 2.85~3.15 | 1.03 | 0.95~1.12 |
| -10 | Survivors | 333.2 | 324.9~342.2 | 1.72 | 1.68~1.77 | 0.74 | 0.7~0.77 |
| -10 | Non-survivors | 142.2 | 133~151.3 | 3.10 | 2.96~3.25 | 1.06 | 0.99~1.14 |
| -9 | Survivors | 335.7 | 327.2~344.3 | 1.70 | 1.65~1.74 | 0.73 | 0.7~0.77 |
| -9 | Non-survivors | 134.7 | 126.9~142.4 | 3.14 | 3~3.27 | 1.08 | 1~1.15 |
| -8 | Survivors | 336.3 | 327.5~344.9 | 1.74 | 1.69~1.8 | 0.73 | 0.7~0.76 |
| -8 | Non-survivors | 133.4 | 125.5~141.1 | 3.18 | 3.04~3.33 | 1.08 | 1.02~1.15 |
| -7 | Survivors | 343.6 | 335.1~352.1 | 1.74 | 1.69~1.79 | 0.72 | 0.69~0.74 |
| -7 | Non-survivors | 133.0 | 124.8~141.6 | 3.19 | 3.06~3.34 | 1.12 | 1.06~1.19 |
| -6 | Survivors | 337.5 | 328.4~346.3 | 1.71 | 1.67~1.77 | 0.73 | 0.7~0.76 |
| -6 | Non-survivors | 126.7 | 118.4~134.7 | 3.26 | 3.12~3.4 | 1.15 | 1.08~1.22 |
| -5 | Survivors | 330.3 | 321.7~338.4 | 1.72 | 1.67~1.77 | 0.72 | 0.69~0.75 |
| -5 | Non-survivors | 123.2 | 115.7~130.2 | 3.32 | 3.18~3.47 | 1.22 | 1.15~1.28 |
| -4 | Survivors | 330.7 | 322.4~339.2 | 1.71 | 1.67~1.76 | 0.71 | 0.69~0.74 |
| -4 | Non-survivors | 121.5 | 113.5~129.4 | 3.42 | 3.27~3.57 | 1.27 | 1.19~1.35 |
| -3 | Survivors | 329.3 | 320.7~337.5 | 1.70 | 1.66~1.76 | 0.70 | 0.67~0.72 |
| -3 | Non-survivors | 114.4 | 107.1~121.9 | 3.61 | 3.44~3.78 | 1.35 | 1.27~1.44 |
| -2 | Survivors | 321.6 | 313.6~329.6 | 1.69 | 1.64~1.74 | 0.70 | 0.67~0.72 |
| -2 | Non-survivors | 114.7 | 107~122.1 | 3.84 | 3.65~4.03 | 1.40 | 1.32~1.49 |
| -1 | Survivors | 323.9 | 315.6~332.6 | 1.67 | 1.62~1.72 | 0.70 | 0.68~0.73 |
| -1 | Non-survivors | 111.9 | 104.3~119.8 | 3.96 | 3.75~4.18 | 1.47 | 1.38~1.56 |
| 0 | Survivors | 358.0 | 349.7~366.1 | 1.64 | 1.59~1.69 | 0.68 | 0.65~0.7 |
| 0 | Non-survivors | 112.0 | 103.5~120.4 | 4.00 | 3.77~4.24 | 1.52 | 1.43~1.62 |

N, number of measurements; CI, confidence interval; TB, total bilirubin; PT, prothrombin time; WBC, white blood cell

B) TB, PT, WBC in Backward

| Days | Group | TB | | PT | | WBC | |
| --- | --- | --- | --- | --- | --- | --- | --- |
|  |  | Fitted value | 95% CI | Fitted value | 95% CI | Fitted value | 95% CI |
| -56 | Survivors | 1.20 | 1.01~1.39 | 13.52 | 13.19~13.84 | 15.9 | 15.1~16.7 |
| -56 | Non-survivors | 0.72 | 0.4~1.04 | 12.81 | 12.24~13.41 | 15.3 | 13.4~17.1 |
| -55 | Survivors | 1.20 | 1.03~1.38 | 13.63 | 13.34~13.89 | 15.6 | 14.9~16.3 |
| -55 | Non-survivors | 0.85 | 0.47~1.25 | 13.82 | 12.2~15.4 | 15.1 | 13.2~16.8 |
| -54 | Survivors | 1.24 | 1.08~1.4 | 13.58 | 13.32~13.82 | 15.6 | 15~16.3 |
| -54 | Non-survivors | 0.83 | 0.3~1.33 | 13.40 | 10.82~16 | 15.0 | 13.3~16.8 |
| -53 | Survivors | 1.14 | 1~1.28 | 13.70 | 13.43~13.98 | 15.5 | 14.9~16.2 |
| -53 | Non-survivors | 0.78 | 0.37~1.18 | 13.04 | 12.16~13.89 | 15.2 | 13.5~17 |
| -52 | Survivors | 1.16 | 1~1.3 | 13.61 | 13.35~13.86 | 15.5 | 14.9~16.1 |
| -52 | Non-survivors | 0.82 | 0.49~1.15 | 13.07 | 12.38~13.76 | 14.9 | 13.2~16.6 |
| -51 | Survivors | 1.18 | 1.02~1.34 | 13.46 | 13.24~13.7 | 15.3 | 14.7~15.9 |
| -51 | Non-survivors | 0.83 | 0.43~1.2 | 13.32 | 12.52~14.12 | 14.6 | 12.9~16.2 |
| -50 | Survivors | 1.16 | 1.04~1.28 | 13.49 | 13.25~13.73 | 15.2 | 14.6~15.7 |
| -50 | Non-survivors | 0.96 | 0.58~1.36 | 13.94 | 12.64~15.29 | 14.5 | 13~16.1 |
| -49 | Survivors | 1.14 | 1.02~1.26 | 13.56 | 13.36~13.77 | 15.0 | 14.5~15.6 |
| -49 | Non-survivors | 0.97 | 0.66~1.3 | 13.39 | 12.7~14.07 | 14.3 | 12.9~15.8 |
| -48 | Survivors | 1.16 | 1.04~1.28 | 13.48 | 13.27~13.68 | 14.9 | 14.4~15.4 |
| -48 | Non-survivors | 0.99 | 0.69~1.3 | 13.24 | 12.59~13.89 | 14.2 | 12.7~15.7 |
| -47 | Survivors | 1.16 | 1.04~1.29 | 13.37 | 13.15~13.57 | 14.8 | 14.3~15.3 |
| -47 | Non-survivors | 0.93 | 0.63~1.25 | 13.94 | 12.86~15.04 | 14.1 | 12.8~15.4 |
| -46 | Survivors | 1.11 | 1~1.23 | 13.49 | 13.28~13.73 | 14.6 | 14.1~15 |
| -46 | Non-survivors | 0.86 | 0.6~1.09 | 14.43 | 13.19~15.76 | 14.1 | 12.9~15.2 |
| -45 | Survivors | 1.17 | 1.07~1.28 | 13.53 | 13.33~13.72 | 14.5 | 14.1~15 |
| -45 | Non-survivors | 0.99 | 0.73~1.25 | 14.25 | 13.33~15.16 | 13.9 | 12.8~15.1 |
| -44 | Survivors | 1.15 | 1.04~1.27 | 13.46 | 13.27~13.64 | 14.3 | 13.9~14.8 |
| -44 | Non-survivors | 0.97 | 0.67~1.26 | 13.98 | 13~14.96 | 14.3 | 13.2~15.4 |
| -43 | Survivors | 1.14 | 1.03~1.25 | 13.38 | 13.2~13.56 | 14.1 | 13.7~14.6 |
| -43 | Non-survivors | 1.24 | 0.97~1.52 | 14.06 | 13.22~14.96 | 14.1 | 13~15.2 |
| -42 | Survivors | 1.12 | 1.02~1.21 | 13.45 | 13.28~13.62 | 14.0 | 13.6~14.4 |
| -42 | Non-survivors | 1.12 | 0.83~1.39 | 14.00 | 13.39~14.6 | 14.2 | 13.2~15.3 |
| -41 | Survivors | 1.11 | 1.01~1.21 | 13.54 | 13.37~13.72 | 13.8 | 13.4~14.2 |
| -41 | Non-survivors | 1.17 | 0.88~1.46 | 13.64 | 13.04~14.24 | 14.1 | 13~15.1 |
| -40 | Survivors | 1.10 | 1~1.19 | 13.58 | 13.39~13.77 | 13.8 | 13.4~14.1 |
| -40 | Non-survivors | 1.21 | 0.93~1.49 | 14.06 | 13.22~14.86 | 14.2 | 13.2~15.2 |
| -39 | Survivors | 1.13 | 1.03~1.23 | 13.54 | 13.36~13.72 | 13.7 | 13.3~14 |
| -39 | Non-survivors | 1.27 | 0.97~1.58 | 13.97 | 13.36~14.55 | 14.3 | 13.3~15.3 |
| -38 | Survivors | 1.07 | 1~1.14 | 13.58 | 13.4~13.75 | 13.8 | 13.4~14.2 |
| -38 | Non-survivors | 1.04 | 0.72~1.35 | 14.24 | 13.44~15.02 | 14.0 | 13.1~15 |
| -37 | Survivors | 1.07 | 0.99~1.15 | 13.43 | 13.23~13.62 | 13.7 | 13.3~14.1 |
| -37 | Non-survivors | 1.01 | 0.72~1.29 | 13.74 | 13.16~14.31 | 14.2 | 13.3~15.2 |
| -36 | Survivors | 1.08 | 1.01~1.15 | 13.58 | 13.38~13.77 | 13.7 | 13.3~14 |
| -36 | Non-survivors | 1.12 | 0.82~1.4 | 14.19 | 13.51~14.83 | 14.1 | 13.3~15 |
| -35 | Survivors | 1.07 | 0.99~1.14 | 13.51 | 13.35~13.68 | 13.5 | 13.2~13.9 |
| -35 | Non-survivors | 1.09 | 0.78~1.38 | 14.39 | 13.73~15.08 | 14.0 | 13.1~14.8 |
| -34 | Survivors | 1.04 | 0.97~1.11 | 13.50 | 13.33~13.67 | 13.5 | 13.1~13.8 |
| -34 | Non-survivors | 1.15 | 0.7~1.57 | 14.29 | 13.65~14.94 | 14.2 | 13.2~15.3 |
| -33 | Survivors | 1.04 | 0.98~1.11 | 13.48 | 13.31~13.65 | 13.4 | 13.1~13.7 |
| -33 | Non-survivors | 1.14 | 0.77~1.52 | 14.30 | 13.85~14.74 | 14.3 | 13.3~15.3 |
| -32 | Survivors | 1.05 | 0.98~1.13 | 13.44 | 13.28~13.6 | 13.3 | 13~13.6 |
| -32 | Non-survivors | 1.13 | 0.78~1.47 | 14.76 | 14.16~15.37 | 14.3 | 13.4~15.3 |
| -31 | Survivors | 1.03 | 0.95~1.1 | 13.37 | 13.2~13.53 | 13.3 | 13~13.6 |
| -31 | Non-survivors | 1.17 | 0.85~1.48 | 14.72 | 14.14~15.26 | 14.2 | 13.3~15.1 |
| -30 | Survivors | 1.02 | 0.93~1.09 | 13.38 | 13.22~13.54 | 13.1 | 12.8~13.4 |
| -30 | Non-survivors | 1.30 | 0.98~1.61 | 14.76 | 14.26~15.27 | 14.1 | 13.1~15 |
| -29 | Survivors | 1.02 | 0.95~1.09 | 13.46 | 13.3~13.62 | 13.1 | 12.8~13.4 |
| -29 | Non-survivors | 1.37 | 1.04~1.68 | 14.84 | 14.29~15.38 | 14.3 | 13.3~15.2 |
| -28 | Survivors | 1.02 | 0.95~1.09 | 13.38 | 13.24~13.53 | 12.9 | 12.6~13.2 |
| -28 | Non-survivors | 1.23 | 0.94~1.51 | 14.61 | 14.17~15.03 | 14.3 | 13.4~15.2 |
| -27 | Survivors | 1.01 | 0.93~1.09 | 13.45 | 13.28~13.62 | 12.9 | 12.7~13.2 |
| -27 | Non-survivors | 1.39 | 1.07~1.7 | 14.75 | 14.25~15.25 | 14.3 | 13.4~15.1 |
| -26 | Survivors | 1.02 | 0.94~1.1 | 13.38 | 13.21~13.54 | 12.8 | 12.6~13.1 |
| -26 | Non-survivors | 1.35 | 1.07~1.62 | 14.82 | 14.38~15.23 | 14.4 | 13.7~15.2 |
| -25 | Survivors | 0.99 | 0.92~1.07 | 13.44 | 13.29~13.58 | 12.6 | 12.3~12.8 |
| -25 | Non-survivors | 1.39 | 1.11~1.65 | 14.87 | 14.4~15.34 | 14.4 | 13.7~15.2 |
| -24 | Survivors | 0.99 | 0.9~1.06 | 13.47 | 13.31~13.62 | 12.6 | 12.4~12.9 |
| -24 | Non-survivors | 1.38 | 1.12~1.64 | 14.97 | 14.58~15.38 | 14.4 | 13.7~15.2 |
| -23 | Survivors | 1.02 | 0.94~1.11 | 13.49 | 13.32~13.64 | 12.4 | 12.2~12.7 |
| -23 | Non-survivors | 1.40 | 1.15~1.64 | 15.16 | 14.76~15.57 | 14.5 | 13.7~15.2 |
| -22 | Survivors | 0.98 | 0.9~1.05 | 13.45 | 13.31~13.6 | 12.3 | 12.1~12.6 |
| -22 | Non-survivors | 1.50 | 1.23~1.76 | 15.07 | 14.63~15.5 | 14.6 | 13.9~15.4 |
| -21 | Survivors | 0.95 | 0.88~1.01 | 13.51 | 13.36~13.65 | 12.2 | 12~12.5 |
| -21 | Non-survivors | 1.47 | 1.22~1.7 | 15.41 | 14.97~15.85 | 14.6 | 13.8~15.3 |
| -20 | Survivors | 1.00 | 0.92~1.07 | 13.45 | 13.3~13.6 | 12.2 | 12~12.4 |
| -20 | Non-survivors | 1.54 | 1.27~1.82 | 15.14 | 14.73~15.55 | 14.7 | 14~15.4 |
| -19 | Survivors | 0.98 | 0.89~1.05 | 13.41 | 13.26~13.57 | 12.1 | 11.9~12.4 |
| -19 | Non-survivors | 1.60 | 1.35~1.86 | 15.18 | 14.77~15.6 | 14.8 | 14.1~15.5 |
| -18 | Survivors | 0.96 | 0.88~1.02 | 13.46 | 13.31~13.61 | 12.0 | 11.8~12.2 |
| -18 | Non-survivors | 1.63 | 1.36~1.89 | 15.54 | 15.17~15.93 | 14.9 | 14.2~15.6 |
| -17 | Survivors | 0.96 | 0.88~1.03 | 13.43 | 13.28~13.6 | 11.9 | 11.7~12.1 |
| -17 | Non-survivors | 1.61 | 1.34~1.89 | 15.67 | 15.25~16.09 | 15.0 | 14.3~15.6 |
| -16 | Survivors | 0.98 | 0.89~1.07 | 13.37 | 13.22~13.54 | 11.8 | 11.6~12 |
| -16 | Non-survivors | 1.72 | 1.44~2 | 15.39 | 14.98~15.81 | 15.2 | 14.5~15.9 |
| -15 | Survivors | 0.94 | 0.86~1.03 | 13.30 | 13.16~13.44 | 11.7 | 11.5~11.9 |
| -15 | Non-survivors | 1.84 | 1.55~2.13 | 15.39 | 14.96~15.78 | 15.4 | 14.8~16.1 |
| -14 | Survivors | 0.90 | 0.84~0.97 | 13.32 | 13.19~13.45 | 11.6 | 11.4~11.8 |
| -14 | Non-survivors | 1.78 | 1.49~2.06 | 15.67 | 15.18~16.13 | 15.6 | 15~16.3 |
| -13 | Survivors | 0.90 | 0.84~0.95 | 13.22 | 13.08~13.36 | 11.5 | 11.3~11.8 |
| -13 | Non-survivors | 1.89 | 1.57~2.19 | 15.38 | 15.04~15.75 | 15.7 | 15~16.3 |
| -12 | Survivors | 0.86 | 0.81~0.92 | 13.26 | 13.11~13.41 | 11.4 | 11.2~11.7 |
| -12 | Non-survivors | 1.82 | 1.53~2.12 | 15.49 | 15.18~15.85 | 15.7 | 15~16.3 |
| -11 | Survivors | 0.88 | 0.81~0.95 | 13.27 | 13.13~13.41 | 11.4 | 11.1~11.5 |
| -11 | Non-survivors | 1.96 | 1.7~2.22 | 15.85 | 15.46~16.23 | 15.9 | 15.3~16.5 |
| -10 | Survivors | 0.88 | 0.8~0.95 | 13.23 | 13.09~13.36 | 11.3 | 11.1~11.5 |
| -10 | Non-survivors | 1.94 | 1.68~2.2 | 15.82 | 15.45~16.18 | 15.9 | 15.3~16.5 |
| -9 | Survivors | 0.88 | 0.8~0.95 | 13.25 | 13.11~13.4 | 11.2 | 11~11.4 |
| -9 | Non-survivors | 1.91 | 1.65~2.16 | 15.92 | 15.58~16.24 | 16.0 | 15.4~16.6 |
| -8 | Survivors | 0.87 | 0.79~0.94 | 13.33 | 13.16~13.51 | 11.2 | 11~11.4 |
| -8 | Non-survivors | 1.93 | 1.65~2.19 | 15.91 | 15.56~16.23 | 16.0 | 15.4~16.6 |
| -7 | Survivors | 0.82 | 0.76~0.88 | 13.26 | 13.12~13.39 | 11.0 | 10.8~11.2 |
| -7 | Non-survivors | 1.96 | 1.71~2.21 | 16.06 | 15.7~16.42 | 16.1 | 15.5~16.7 |
| -6 | Survivors | 0.88 | 0.8~0.95 | 13.16 | 12.99~13.33 | 11.1 | 10.9~11.3 |
| -6 | Non-survivors | 2.04 | 1.78~2.3 | 16.01 | 15.69~16.34 | 16.1 | 15.6~16.7 |
| -5 | Survivors | 0.86 | 0.8~0.93 | 13.08 | 12.94~13.21 | 11.0 | 10.8~11.2 |
| -5 | Non-survivors | 2.07 | 1.82~2.33 | 16.16 | 15.86~16.49 | 16.1 | 15.6~16.7 |
| -4 | Survivors | 0.84 | 0.78~0.9 | 13.10 | 12.95~13.24 | 10.9 | 10.7~11.1 |
| -4 | Non-survivors | 2.14 | 1.89~2.4 | 16.30 | 15.94~16.65 | 16.1 | 15.5~16.6 |
| -3 | Survivors | 0.81 | 0.75~0.86 | 12.99 | 12.87~13.11 | 10.8 | 10.7~11 |
| -3 | Non-survivors | 2.25 | 1.98~2.51 | 16.58 | 16.21~16.99 | 16.2 | 15.6~16.7 |
| -2 | Survivors | 0.82 | 0.78~0.86 | 12.96 | 12.83~13.07 | 10.9 | 10.7~11 |
| -2 | Non-survivors | 2.31 | 2.06~2.57 | 16.94 | 16.48~17.38 | 16.5 | 15.9~17 |
| -1 | Survivors | 0.82 | 0.78~0.87 | 12.88 | 12.75~13.01 | 10.8 | 10.7~11 |
| -1 | Non-survivors | 2.38 | 2.1~2.65 | 17.31 | 16.8~17.85 | 16.6 | 16~17.1 |
| 0 | Survivors | 0.77 | 0.72~0.82 | 12.89 | 12.78~13 | 10.5 | 10.3~10.6 |
| 0 | Non-survivors | 2.48 | 2.18~2.76 | 17.44 | 16.9~18.02 | 16.5 | 15.9~17.1 |

N, number of measurements; CI, confidence interval; TB, total bilirubin; PT, prothombin time; WBC, white blood cell
